# *Lrig1*-expressing quiescent stem cells maintain vocal fold mucosal homeostasis via *Notch* signaling

**DOI:** 10.1073/pnas.2513590122

**Published:** 2025-11-25

**Authors:** Vlasta Lungova, Jessica M. Fernandez, Yujia Cai, Christina Kendziorski, Susan L. Thibeault

**Affiliations:** ^a^Department of Otolaryngology Head and Neck Surgery, University of Wisconsin Madison, Madison, WI 53705; ^b^Department of Biostatistics and Medical Informatics, University of Wisconsin Madison, Madison, WI 53705

**Keywords:** vocal folds, larynx, stem cells, homeostasis, quiescence

## Abstract

Identifying progenitor cells in laryngeal and vocal fold (VF) mucosa is essential for advancing stem cell–based therapies for VF diseases. In this study, we used *Lrig1* (leucine-rich repeats and immunoglobulin-like domains 1) gene to label tissue-resident stem cells within laryngeal and VF mucosa, aiming to investigate cellular networks underlying VF homeostasis and regeneration. *Lrig1*^+^ cells exhibited hallmark features of quiescent stem cells—including slow cycling, long-term persistence, and differentiation potential—and contributed to sustained VF regeneration and repair after naphthalene injury. By one week post injury *Lrig1*^+^ progeny restored the damaged VF epithelium. Transcriptionally, these cells suppressed cell type specific genes and proliferation while activating programs related to RNA metabolism, epigenetic regulation, and protein ubiquitination, consistent with quiescence and readiness for activation. *Lrig1*^+^ cells were enriched in basal, parabasal, and immature secretory populations within surface epithelium and submucosal glands, residing in niches characterized by transcriptional flexibility and spatial shielding from direct stress—features that likely prevent premature or chronic mobilization. Notably, analogous LRIG1 expressing cells were identified in human laryngeal and VF tissues, highlighting the translational relevance of our findings. To explore regulation of *Lrig1*^+^ cell quiescence, we conditionally deleted *Notch1,* which led to epithelial hyperplasia, expansion of secretory populations, and mucus hyperproduction. In summary, *Lrig1* marks a conserved, quiescent stem cell population in the larynx and VFs that supports long-term tissue homeostasis and repair, with quiescence maintained via *Notch1* signaling.

## Significance

*Lrig1* marks a conserved, quiescent stem cell population in laryngeal and vocal fold (VF) mucosa, essential for maintaining tissue homeostasis and repair after injury. These cells are associated with less differentiated cell types; they are positioned in locations protected from direct exposure to stress factors. Transcriptionally, they suppress cell type–specific genes and proliferation, while activating a global transcriptional program related to RNA metabolism, epigenetic regulation and protein ubiquitination—supporting quiescence and readiness for activation. Their quiescence is regulated via *Notch1* signaling. Disruption of this pathway leads to pathological epithelial remodeling and mucus hyperproduction. These findings enhance our understanding of laryngeal tissue homeostasis and offer a foundation for investigating how stem cell dysregulation may contribute to laryngeal and VF diseases.

VF mucosa is subjected to intense mechanical stress during phonation, as it undergoes rapid and repetitive vibratory cycles that can reach frequencies of many hundred times per second (1). These vibrations generate significant shearing forces, collision impact forces, and tissue deformation, particularly at the epithelial surface and within the underlying lamina propria. VF mucosa is exposed to environmental and chemical insults, further compromising tissue integrity (2). Together, these challenges necessitate mechanisms for tissue maintenance, repair, and regeneration to preserve vocal function. Tissue maintenance and regeneration rely on a population of cells with regenerative potential that possess the capacity for self-renewal and differentiation, supporting physiological turnover and facilitating tissue repair after injury. Under normal homeostatic conditions, these cells typically remain quiescent, and slow-cycling retaining DNA label, but become activated in response to damage or stress (3). Stem cell quiescence is an actively regulated state, maintained by signaling pathways that keep cells poised for rapid activation while preventing premature differentiation and/or exhaustion (4).

The identification of stem/progenitor cells within laryngeal and VF mucosa has become an emerging area of interest, with significant implications for tissue engineering and the development of stem cell–based therapies to treat various VF diseases (5). In the epithelium, slow-cycling BrdU-retaining epithelial cells have been identified in murine VFs (6), scattered across the basal and parabasal layers of the stratified squamous epithelium. Cells in the basal layer are generally considered the least differentiated (7) and exhibit stem cell–like properties (8), while parabasal cells represent a transitional population that actively regulates both the proliferation and maintenance of upstream basal cells as well as differentiation of downstream progeny (9). Despite insights into candidate progenitor populations, direct evidence linking them to mucosal maintenance, repair, and pathological mucosal remodeling remains limited. Such remodeling—characterized by epithelial hyperplasia and/or an increased mucus—is commonly observed in clinical contexts, including chronic inflammatory conditions and VF lesions (10–12).

In this study, we used the *Lrig1* (leucine-rich repeats and immunoglobulin-like domains 1) gene to label tissue-resident stem cells, within the laryngeal and VF mucosa. *Lrig1* encodes a transmembrane protein that has been extensively studied in epithelial stem cell niches across various organs, where it plays a key role in maintaining stem cell quiescence and regulating proliferation during homeostasis and in response to injury or mechanical stress (13). We hypothesize that *Lrig1*-expressing cells constitute a conserved population of quiescent stem cells in the larynx and VFs, playing a critical role in long-term tissue homeostasis and repair. Dysregulation of their quiescent state triggers pathological changes in the mucosa.

To investigate the role of *Lrig1* expressing cells in laryngeal and VF tissues, we combined lineage tracing in *Lrig1Cre^ERT2/+^; ROSA^LSL-tdTomato/+^* reporter mice with single-cell RNA sequencing (scRNA-seq) of flow cytometry-sorted *tdTomato*^+^ (*tdTom*) cells. This approach enabled spatial characterization and transcriptional profiling of *Lrig1* cells. Moreover, we performed naphthalene (NA) injury to damage the epithelium and assess regenerative contribution of *Lrig1*-expressing cells. Last, to understand the mechanism regulating *Lrig1* cell quiescence, we examined the role of *Notch1* signaling, as the *Notch* pathway has been shown to regulate progenitor activation and cell fate decisions in a cell-autonomous manner (14). Conditional deletion of *Notch1* in *Lrig1*-expressing cells primarily affected epithelial compartments, leading to an expansion of basal cells in laryngeal and VF epithelium, along with an increase in secretory cell populations. This resulted in epithelial hyperplasia, VF epithelial thickening, and excessive mucus production—changes likely to impair VF function and compromise airway patency. In summary, *Lrig1* marks a conserved dormant population of stem/progenitor cells in the larynx and VFs, with quiescence regulated by *Notch1* signaling.

## 1. Results

### 1.1. Lineage Tracing of *tdTom*-labeled *Lrig1* Cells Within Laryngeal Epithelium.

**Fig. 1.**
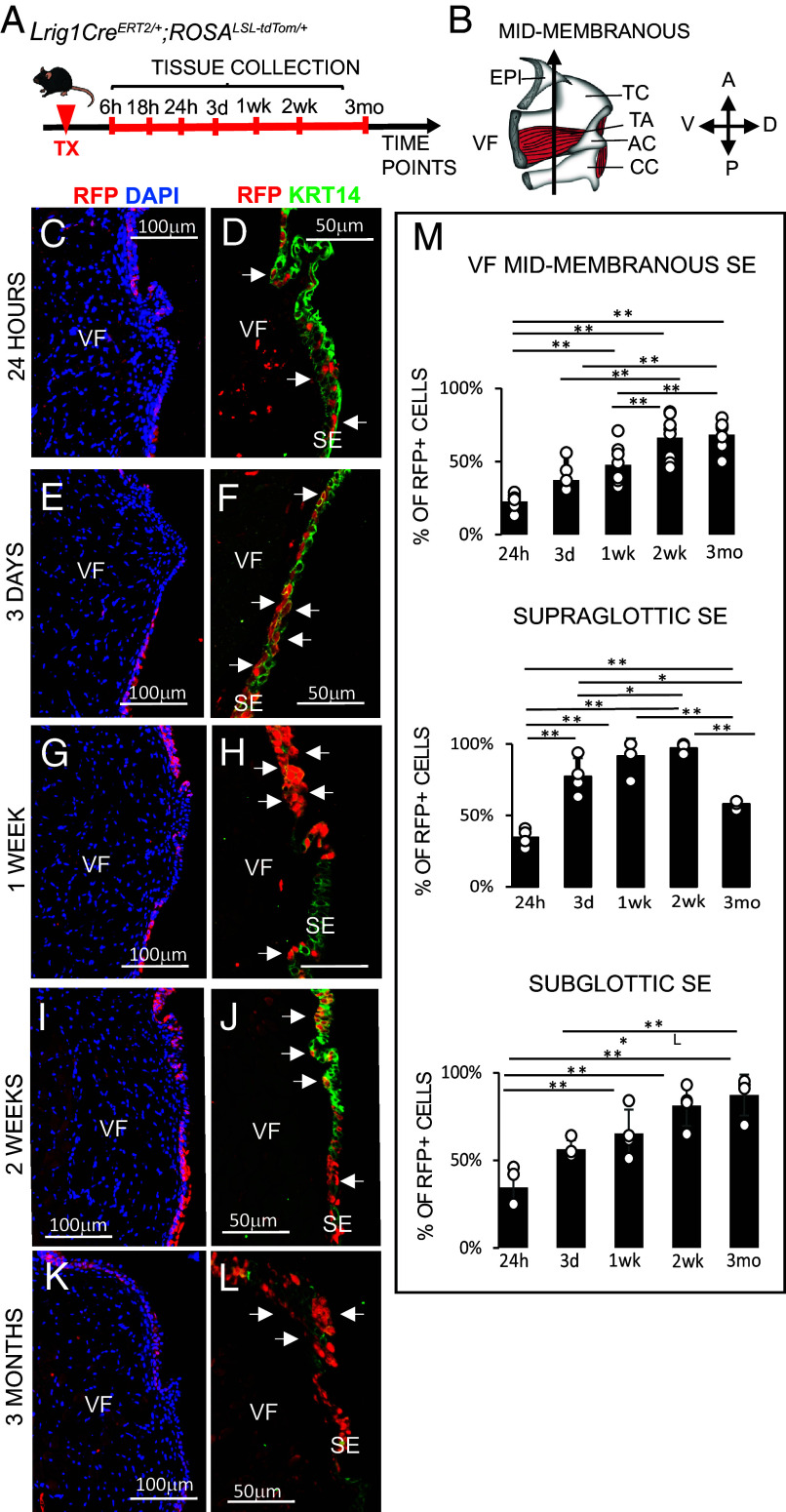
Lineage tracing using *tdTom* reporter in *Lrig1^CreERT2/+^* mice. (*A*) Schematics of experimental model and strategy. (*B*) Sagittal schematic of murine larynx showing mid-membranous VF and key laryngeal structures. Directional axes are indicated. (*C*) IF for anti-RFP (red) and DAPI in VFs 24 h PR. (*D*) IF for anti-RFP (red) and anti-KRT14 (green) showing distribution of RFP+ cells in KRT14+ epithelium. (*E*) IF for anti-RFP (red) and DAPI in VFs 3d PR. (*F*) IF for anti-RFP (red) and anti-KRT14 (green) showing distribution of RFP+ cells in KRT14+ epithelium. (*G*) IF for anti-RFP (red) and DAPI in VFs 1wk PR. (*H*) IF for anti-RFP (red) and anti-KRT14 (green) showing distribution of RFP+ cells in KRT14+ epithelium. (*I*) IF for anti-RFP (red) and DAPI in VFs 2wk PR. (*J*) IF for anti-RFP (red) and anti-KRT14 (green) showing distribution of RFP+ cells in KRT14+ epithelium. (*K*) IF for anti-RFP (red) and DAPI in VFs 3mo PR. (*L*) IF for anti-RFP (red) and anti-KRT14 (green) showing distribution of RFP+ cells in KRT14+ epithelium. White solid arrows in the panels of *D*, *F*, *H*, *J*, and *L* denote RFP+ cells in basal and parabasal cell layers in the SE. (*M*) Percentage of RFP+ cells in SE of VF mid-membranous region, supraglottis, and subglottis. Error bars with individual data points represent ± SEM. One-Way ANOVA of variance for independent or correlated samples statistical analysis along with the Plus Tukey HSD test were used to confirm statistical significance, *P ≤ 0.05 and **P ≤ 0.01. Abbreviations: A, anterior; AC, arytenoid cartilage; CC, cricoid cartilage; d, day; D, dorsal; mo, month; P, posterior; RFP, red fluorescent protein; SE, surface epithelium; SMG, submucosal glands; TC, thyroid cartilage; TA, thyroarytenoid muscle; TX, tamoxifen; V, ventral; VF, vocal fold; wk, week.

To assess the presence and fate of *Lrig1*-expressing cells in the larynx and VFs during homeostasis, we generated *Lrig1Cre^ERT2/+^; ROSA^LSL-tdTom/+^* reporter mice with *Lrig1*^+^ cells labeled with red fluorescent protein (RFP), upon tamoxifen (TX)-induced Cre activation. We used a single TX injection to activate recombinase in reporter mice (Fig. 1*A*). *Lrig1Cre^ERT2/+^; ROSA^LSL-tdTom/+^* without TX injection were controls (*SI Appendix*, Fig. S1 *D*–*G*). Whole larynges from control and reporter mice were harvested at multiple time points—6 h, 18 h, 24 h, 3 d, 1 wk, 2 wk, and 3 mo post-recombination (PR) (Fig. 1*A*). Larynges were processed and sectioned coronally; analyses focused on the mid-membranous VF region (Fig. 1*B* and *SI Appendix*, Fig. S1 *A*–*C*), given its well-established role in tissue oscillation in humans (15).

Our lineage tracing data demonstrate that RFP signaling was absent at 6 h PR but appeared in laryngeal and VF mucosa by 18 h PR (*SI Appendix*, Fig. S1 *H*–*K*), with a few RFP^+^ cells at the VF surface epithelium (SE), supraglottic and subglottic SE, and submucosal glands (SMGs). At 24 h PR, approximately 23% of RFP^+^ cells were detected in the SE of the VF (Fig. 1 *C*, *D*, and *M*), and were localized to basal and parabasal compartments (Fig. 1*D*). In the supraglottic and subglottic areas 24 h PR, RFP^+^ cells accounted for 35 and 40% of the SE respectively, with only sporadic labeling in SMGs (Fig. 1*M* and *SI Appendix*, Fig. S2 *A* and *B*). By 3d PR, the proportion of RFP^+^ cells increased across all regions, including SE and SMG (Fig. 1 *E* and *F* and *SI Appendix*, Fig. S2 *C* and *D*), with a significant rise only in the supraglottic SE (*P* = 0.009958) (Fig. 1*M*). At 1 and 2wk PR, RFP+ cells populated nearly the entire SE of the VF, supraglottis, subglottis, and SMGs, with their preferential localization in the basal and parabasal epithelial layers (Fig. 1 *G*–*J* and *SI Appendix*, Fig. S2 *E*–*H*). By 2wk PR, percentage of RFP+ cells had significantly increased to 66% in VF SE (*P* = 0.00000001747), 97% in supraglottic (*P* = 0.0000003238), and 81% of subglottic SE (*P* = 0.0002119) (Fig. 1*M*). RFP signal persisted in all regions 3 mo PR (Fig. 1 *K*–*M* and *SI Appendix*, Fig. S2 *I* and *J*). Moreover, we observed that RFP^+^ clones moved into suprabasal cell layers (Fig. 1*L* and *SI Appendix*, Fig. S2*I*) while being replaced by new cells from the basal compartment. In the supraglottis, cell replacement likely contributed to the significant decrease in percentage of RFP^+^ cells observed at 3mo PR compared to earlier time points (*P* = 0.0001002) (Fig. 1*M*). Besides epithelium, RFP^+^ signal emerged in the thyroarytenoid (TA) muscle (Fig. 1 *I* and *K*), indicating that *Lrig1* cells reside beyond the epithelial layer.

### 1.2. Characterization of *tdTom*-Labeled *Lrig1* Cells in SE and SMG Populations.

Next, we examined epithelial cell populations harboring *tdTom*-labeled *Lrig1* cells. As mentioned above, immunofluorescent (IF) staining confirmed that the majority of *Lrig1* cells were epithelial, as demonstrated by the colocalization of RFP with KRT14 (*SI Appendix*, Fig. S3 *A* and *B*). RFP-positive cells were located in basal and parabasal P63+ cell layers (*SI Appendix*, Fig. S3 *B* and *C*), but interestingly, not all P63+ or KRT14+ epithelial cells simultaneously expressed RFP. While a subset of RFP cells was proliferative, as indicated by Ki67 expression (*SI Appendix*, Fig. S3*D*), the majority of Ki67+ cells were RFP negative, consistent with the literature reporting low proliferation rates in quiescent *Lrig1+* cell populations (16). Notably, RFP cells did not overlap with mature secretory club cells, stained with anti-SCGB1A1, in SE and SMGs (*SI Appendix*, Fig. S3 *E*–*G*). Within SMGs, some RFP cells colocalized with aSMA positive myoepithelial cells (*SI Appendix*, Fig. S3 *H* amd *I*). We observed that some RFP-positive SMG likely interacted with SE, either through direct incorporation, as seen in the supraglottis (*SI Appendix*, Fig. S3 *J* and *K*), or via ductal connections in the subglottis (*SI Appendix*, Fig. S3*L*). Notably, glands located deeper within the tissue showed lower KRT14 expression compared to those situated closer to the epithelium (*SI Appendix*, Fig. S3 *J* and *K*). Together, these findings support our hypothesis that *Lrig1* preferentially labels quiescent cells and is associated with less differentiated epithelial cell types.

### 1.3. Characterization of LRIG1 Cells in Human Larynx and VF.

**Fig. 2.**
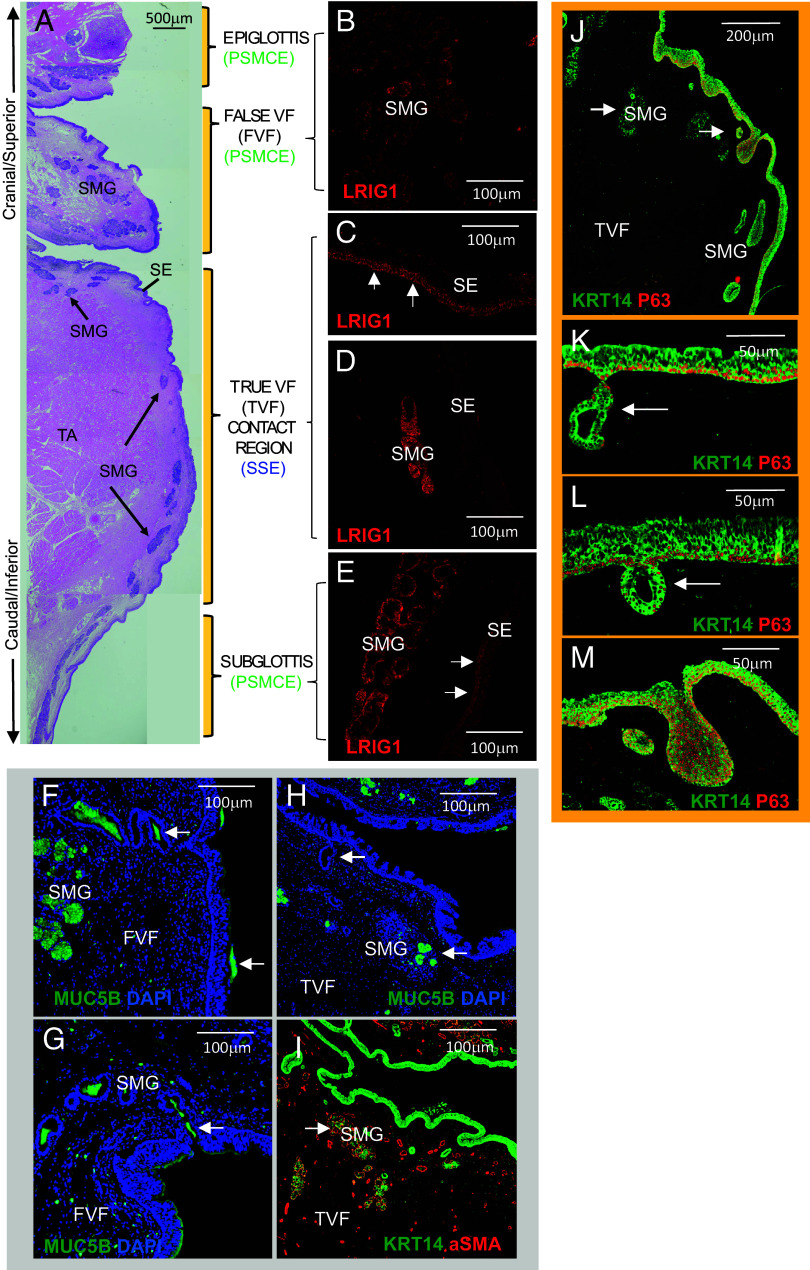
LRIG1+ cells in human larynx and VFs. (*A*) H&E coronal mid membranous human larynx shows epiglottis, FVF, and TVF with distinct epithelial linings. (*B*–*E*) IF for anti-LRIG1 (red) in SMGs in FVFs and along the superior margin of TVFs (*B* and *C*), VF contact region (*D*) and subglottis (*E*). (*F*–*H*) Anti-MUC5B staining in SMGs in FVFs (*F* and *G*) and along the superior margin of TVFs (*H*). Solid white arrows denote MUC5B^+^ cells a dashed white arrow indicates and SMGs lacking MUC5B expression. (*I*) Costaining for αSMA and KRT14 in the superior margin of the TVFs. Solid white arrows denote αSMA^+^/KRT14^+^ double-positive SMG cells. (*J*–*M*) Costaining for P63 and KRT14 in the superior margin of the TVF. A solid white arrow denotes P63^+^/KRT14^+^ double-positive SMG cells located near the SE, and weak P63^+^/KRT14^+^ staining in SMGs deeper within the tissue. Abbreviations: PSMCE, pseudostratified mucociliary epithelium; SE, surface epithelium; SMG, submucosal glands; SSE, stratified squamous epithelium; FVF, false vocal folds; TA, thyroarytenoid muscle; TVF, true vocal folds.

To investigate whether similar LRIG1+ cell populations exist in the human larynx and VFs, we harvested tissue from adult human larynges. We first performed H&E staining to provide an anatomical orientation of the larynx, and corresponding epithelial surfaces, stratified squamous epithelium (SSE) in true VFs (TVF) and pseudostratified mucociliary epithelium (PSMCE) in the false VFs (FVF), epiglottis, and subglottis (Fig. 2*A*). As expected, SMGs were primarily located in the epiglottis and FVF, with additional glands observed along the superior TVF margin and in the subglottis (Fig. 2*A*). Subsequent anti-LRIG1 staining revealed negative LRIG1 staining in FVF SMGs, which is consistent with their primary secretory function (Fig. 2 *B*, *F*, and *G*). LRIG1 positive cells were found in SE lining the superior TVF margin, where it was detected in the basal epithelial layers (Fig. 2*C*) and SMGs that extended into the VF contact zone (Fig. 2*D*). Notably, signal diminished within SE basal layers of the VF contact zone but re-emerged in the subglottis (Fig. 2 *D* and *E*), where it was also detected in SMGs (Fig. 2*E*).

We investigated potential interactions between SMGs and SE by staining for MUC5B, P63, and KRT14 (Fig. 2 *F*–*M*). Our data show that MUC5B^+^ SMGs interacted with epithelium, contributing to surface lubrication (Fig. 2 *F*–*H*). Interestingly, while glands in the FVF primarily expressed MUC5B, glands in the superior VF margin showed partial MUC5B expression, and no MUC5B expression was detected in glands adjacent to the SE, suggesting a potential dual role for these glands (Fig. 2*H*). Deeper glands contained aSMA^+^ myoepithelial cells and exhibited lower expression of KRT14/P63 compared to those near SE (Fig. 2 *I* and *J*). Additional evidence supporting incorporation of SMGs into SE was provided by anti-P63 and anti-KRT14 staining (Fig. 2 *K*–*M*). Together, these findings suggest that LRIG1^+^ cells in the human larynx and VFs may support long-term mucosal homeostasis and repair, underscoring the translational relevance of our work.

### 1.4. Molecular Profiling of Murine *Lrig1*^+^ and *Lrig1^−^* Cell Populations.

#### 1.4.1. Pseudobulk differential gene expression and gene set enrichment analysis of *Lrig1*^+^ and *Lrig1^−^* cell populations.

To validate the stem/progenitor-like phenotype of *Lrig1^+^* cells in the murine larynx/VFs, we performed single-cell RNA sequencing (scRNA-seq) on flow cytometry-sorted *tdTom—*positive cells (*SI Appendix*, Fig. S4). In line with our lineage tracing experiments, we first generated *Lrig1Cre^ERT2/+^; ROSA^LSL-tdTom/+^* reporters, then activated Cre using two TX injections for two consecutive days and collected tissues one day PR. Larynges were then dissociated, and cells were sorted based on *tdTom*^+^ signal (*SI Appendix*, Fig. S4*A*). Wild-type (WT) and *ROSA^LSL-tdTom/+^* larynges were used as negative and background controls, respectively (*SI Appendix*, Fig. S4*B*). To obtain sufficient number of *tdTom*^+^ cells for scRNA-seq, we pooled 10 larynges per biological replicate (n = 10 per replicate; 5 males and 5 females). In total, three biological replicates (BR) were used.

Following quality control (*SI Appendix*, Fig. S4 *C* and *D*), a total of 45,680 cells were recovered, with 49.6% (22,627 cells) classified as *Lrig1^+^* (*Lrig1* expression > 0), and 50.4% (23,053 cells) as *Lrig1^−^* (*Lrig1* expression = 0, below detection threshold). Uniform Manifold Approximation and Projection (UMAP) using Seurat package (v5.1.0) shows *Lrig1^+^* and *Lrig1^−^* cells were distributed across the same cell populations (*SI Appendix*, Fig. S5*A*), suggesting that differences in *Lrig1* expression may reflect varying activation states rather than distinct cell types. This interpretation was further supported by pseudobulk differential gene expression (DEG) analysis between *Lrig1^+^* and *Lrig1****^−^*** groups, which revealed that *Lrig1*^+^ cells significantly downregulated many cell type–specific genes—including *Acta2, Myh1, S100a9, Tagln, Dcpp2,* and *Il1b*—compared to *Lrig1^−^* cells, consistent with a quiescent transcriptional state (*SI Appendix*, Fig. S5*C*). Additionally, *Lrig1^+^* cells exhibited higher overall RNA counts (*SI Appendix*, Fig. S5*B*), which could be attributed to technical aspects of the sequencing method or, alternatively, to underlying biological differences between the two groups. Biologically, quiescent stem cells often retain higher RNA content due to their larger size and distinct metabolic state (4).

Gene set enrichment analysis (GSEA) confirmed that silencing of cell type–specific genes in *Lrig1*^+^ cells is accompanied by the activation of a global transcriptional program related to RNA metabolism, epigenetic regulation and protein ubiquitination (*SI Appendix*, Fig. S6). We performed GSEA comparing *Lrig1*^+^ and *Lrig1^−^* populations (*SI Appendix*, Fig. S6), as well as comparing *Lrig1^High^* cells (with expression levels above the median *Lrig1* expression) and *Lrig1^Low^* cells (with downregulated *Lrig1* expression equal to and below the median threshold) (*SI Appendix*, Fig. S7). In *Lrig1^+^* and *Lrig1^High^* groups, RNA metabolism–related processes encompassed both coding and noncoding RNAs, including their processing, modification, degradation, and the biogenesis of ribonucleoprotein complexes. Epigenetic regulatory mechanisms—including DNA methylation, heterochromatin formation, and chromatin remodeling—were particularly prominent in the *Lrig1^High^* group (*SI Appendix*, Fig. S7), along with gene sets related to stem cell population maintenance, regulation of cell number, and protein ubiquitination. These processes are hallmarks of stem cell quiescence and supportive mechanisms of stem cell function (4).

We observed that *Lrig1*^+^ and *Lrig1^High^* cells were enriched for gene expression programs related to synapses, and axonogenesis suggesting potential interactions with the autonomic nervous system. Such neural regulation has been reported to modulate stem cell activation in hair follicle (17) and intestinal stem cells (18). In contrast, *Lrig1^−^* and *Lrig1^Low^* cells activated gene programs primarily linked to their functional roles, including, ECM organization, innate immune responses, and regulation of angiogenesis. In addition, they upregulated proliferation-related pathways, such as microtubule bundle formation (*SI Appendix*, Fig. S7), indicating a transition from quiescence toward differentiation, maturation, and proliferation.

#### 1.4.2. Cell type identification and transcriptional profiling of *Lrig1^+^* and *Lrig1^−^* cells.

**Fig. 3.**
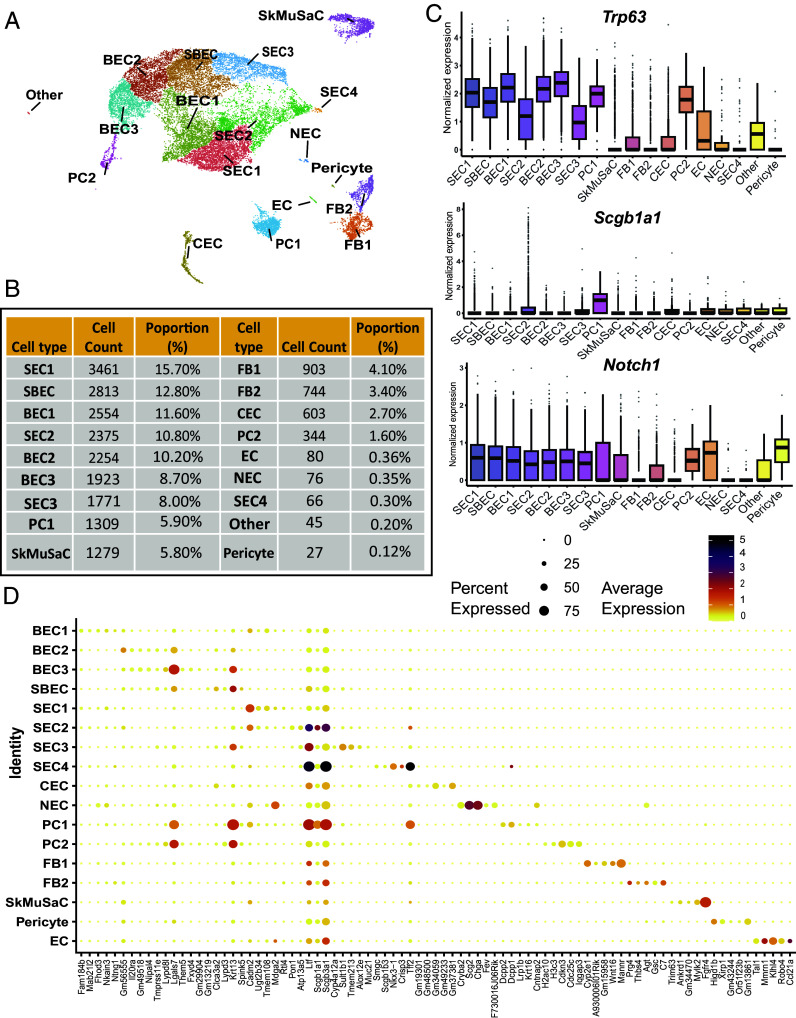
Main cell types and gene expression for *Lrig1*^+^ populations. (*A*) UMAP visualization of the *Lrig1*^+^ cell population. (*B*) Summary table of cell types identified in *Lrig1*^+^ population, including total cell counts and exact proportions for each cell type. (*C*) Box plots displaying expression levels of *Trp63*, *Scgb1a1*, and *Notch1* across all identified cell types. (*D*) Dot plot showing top 5 differentially expressed genes that define each cluster. Dot size represents percentage of cells expressing the gene in each cluster, and color scale indicates average gene expression levels. Abbreviations: BEC, basal epithelial cell; CEC, ciliated epithelial cell; EC, endothelial cell; FB, fibroblast; NEC, neuroendocrine cell; PC, proliferating cell; SEC, secretory epithelial cell, SBEC, suprabasal epithelial cell; SkMuSaC, skeletal muscle satellite cell.

**Fig. 4.**
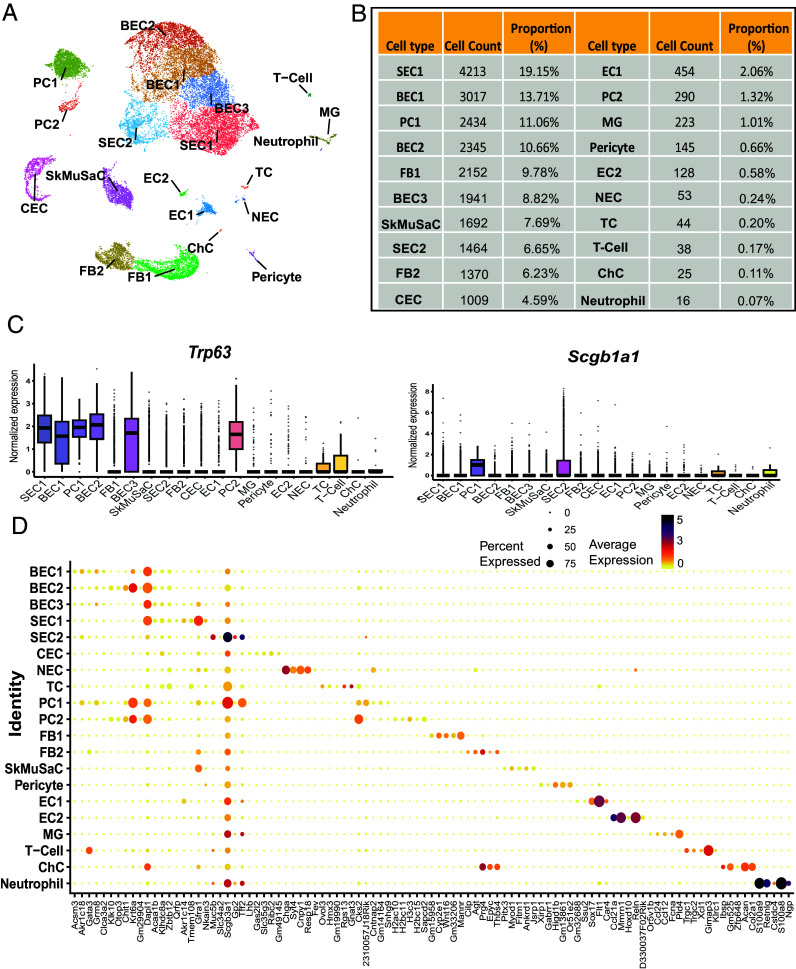
Main cell types and gene expression for *Lrig1*^−^ populations. (*A*) UMAP visualization of the *Lrig1*^−^ cell population. (*B*) Summary table of all cell types identified within the *Lrig1*^−^ population, including total cell counts and exact proportions. (*C*) Box plots displaying expression levels of *Trp63*, and *Scgb1a1* across all identified cell types. (*D*) Dot plot showing top 5 differentially expressed genes that define each cluster. Dot size represents percentage of cells expressing the gene in each cluster, and color scale indicates average gene expression levels. Abbreviations: BEC, basal epithelial cell; CEC, ciliated epithelial cell; ChC, chondroblast; EC, endothelial cell; FB, fibroblast; MG, macrophage; NEC, neuroendocrine cell; PC, proliferating cell; SEC, secretory epithelial cell, SBEC, suprabasal epithelial cell; SkMuSaC, skeletal muscle satellite cell; TC, tuft cell.

We applied unsupervised clustering to *Lrig1*^+^ and *Lrig1*^−^ groups and generated UMAPs for each population (Figs. 3 and 4). In the *Lrig1^+^* group, we identified 18 distinct cell types (Fig. 3 *A* and *B*) which preferentially included epithelial cell populations, such as basal epithelial cells 1, 2, and 3 (BEC1, BEC2, and BEC3); suprabasal epithelial cells (SBEC); secretory epithelial cells 1, 2, 3, and 4 (SEC1—club cell 1, SEC2—club cell 2, SEC3—goblet cell, and SEC4—serous cells in SMGs); ciliated epithelial cells (CEC) and neuroendocrine cells (NEC). Then, we identified two proliferating cell populations (PC1 and PC2), and nonepithelial cell types, such as fibroblasts 1 and 2 (FB1, and FB2), endothelial cells (EC), and skeletal muscle satellite cells (SkMuSaC), pericyte (smooth muscle cell, SmMuC), and others. In terms of cell counts and proportions, *Lrig1*^+^ cells were most abundant in the SEC1 (3,461 cells; 15.70%) and SBEC (2,813 cells; 12.80%) (Fig. 3*B*). SEC1 cells likely represent an immature secretory population, characterized by high expression of *Trp63*—a marker of early-stage secretory cells—and low expression of *Scgb1a1*, which marks mature secretory differentiation (Fig. 3*C*). SBEC showed high *Trp63* expression, consistent with a parabasal epithelial identity (Fig. 3*C*). Other highly represented *Lrig1*^+^ populations included basal epithelial cells—BEC1 (2,554 cells, 11.6%), BEC2 (2,254 cells, 10.2%), and BEC3 (1,923 cells, 8.7%). On the other hand, *Lrig1^+^* expressing PCs and FBs were less abundant and highly specialized epithelial populations such as CEC, SEC4, and NEC, were relatively rare (Fig. 3*B*), We also evaluated expression of *Notch1* in *Lrig1*^+^ cells, given its role in regulating epithelial cell fate (19). We found that all less differentiated epithelial cell types, along with PCs, exhibited high *Notch1* expression (Fig. 3*C*).

Looking deeper into cell identities, we examined gene expression patterns across all *Lrig1*^+^ clusters by generating a Dot-Plot of the top five differentially expressed genes (DEGs) per cluster (Fig. 3*D*). In this graph, we excluded the cluster annotated as “Other” due to its negligible abundance. Overall, *Lrig1*^+^ populations exhibited low expression of lineage-defining genes. Many of the top DEGs began with “Gm-” or ended with “-Rik”—corresponding to protein-coding RNAs, long noncoding RNAs, and antisense transcripts consistent with enhanced RNA metabolism (https://useast.ensembl.org/). Among cell-identity-associated genes, we observed slight upregulation of *Lgals7* and *Krt13*, which were enriched in BEC2, BEC3, SBEC, and PC clusters, suggesting an association with VF SSE. Secretoglobin family genes, including *Scgb1a1*, *Scgb3a1*, and *Ltf*, were prominent in secretory epithelial clusters, with SEC4 representing the most mature secretory state. NEC showed expression of lineage-specific markers *Scg2* and *Chga*, encoding secretogranin and chromogranin, respectively. PC1 and PC2 likely represented a mixed population of dividing cells (Fig. 3 *C* and *D*).

In the *Lrig1^−^* group, we identified 20 distinct cell types (Fig. 4*A*), including epithelial cells—BEC1–3, SEC1 (club cells), SEC2 (goblet cells)—and specialized types—NEC, CEC, and newly identified Tuft cells (TC), also known as brush cells. We identified PC1 and PC2 and nonepithelial populations, including FB1, FB2, chondroblasts, endothelial cells (EC1—blood vessels; EC2—lymphatic vessels), skeletal and smooth muscle-associated cells (SkMuSaC, pericyte), and immune cells—macrophages (MG), T-cells, and neutrophils. In terms of differentiation, SEC1 expressed high levels of *Trp63* and low levels of *Scgb1a1*, indicating an early stage of differentiation, while SEC2 downregulated *Trp63* and upregulated *Scgb1a1*, consistent with a more mature phenotype (Fig. 4*C*). Cell counts demonstrated the highest numbers in SEC1 (4,213 cells, 19.15%), BEC1 and BEC2 (3,017 and 2,345 cells, 13.71 and 10.66%, respectively), and PCs (2,434 cells, 11.06%). We observed a significant increase in nonepithelial populations, especially FBs (FB1:2152 cells, 9.78%; FB2:1370 cells, 6.23%) (Fig. 4*B*).

A Dot-Plot for the *Lrig1*^−^ group confirmed activation and increased transcriptional activity of cell identity genes across most cell types, particularly those with specialized functions, such as SEC2, NEC, TC, EC, chondroblasts, and immune cells (Fig. 4*D*). In FBs, we identified a clear distinction between *Wnt16*-expressing FB1 and FB2, which expressed chondroblast associated markers, suggesting that FB2 may be localized in the perichondrium. In less differentiated epithelial populations—BEC1, BEC2, BEC3, SEC1, and proliferating cells—we observed upregulation of genes like *Krt6a* and *Dapl1*. The *Dapl1* gene is predicted to enable ribosome binding activity and acts upstream of or within proliferation and differentiation https://www.ncbi.nlm.nih.gov/gene/76747. In the context of *Lrig1*^−^ cells, *Dapl1* upregulation may reflect enhanced growth and differentiation, suggesting a shift toward active proliferation and maturation, even within less differentiated cells.

#### 1.4.3. Developmental trajectories within *Lrig1^+^* cell populations.

To explore dynamic progression of epithelial cell states within *Lrig1*^+^ cells, we performed pseudotime analysis to reconstruct developmental trajectories (*SI Appendix*, Fig. S8). We first assessed the SSE lineage, following the BEC3 → BEC2 → SBEC → SEC3 trajectory (*SI Appendix*, Fig. S8 *A* and *B*), which reflected cell hierarchy observed in the VF epithelium (7). The identity of the SSE lineage was further supported by the expression of genes associated with SSE identity and differentiation, including *Tmprss11a, Krt14, Tmprss11b, and Krt13* (20) (*SI Appendix*, Fig. S8*E*). Next, we focused on the pseudostratified epithelial lineage, beginning with BEC1 → SEC1 (club1) → SEC2 (club2) or BEC1 → SEC1 (club1) → SEC4 (goblet), which captures the progression of major cell types within the pseudostratified epithelium (*SI Appendix*, Fig. S8 *C* and *D*). CEC and NEC formed distinct clusters; indicative of unique transcriptional programs associated with their specialized functions. TC were exclusively detected in the *Lrig1^−^* population. This pseudostratified lineage and associated secretory phenotype were further supported by the expression of airway epithelial markers such as *Nkx2-1, Scgb3a2, Aldh1a7, and Scnn1a* (20) (*SI Appendix*, Fig. S8*F*). Expression of *Dmbt1*, a marker of SMGs, further suggests that SEC4 may correspond to serous cells within these glands, as they also express *Ltf, Muc5b*, and *Scgb3a1* (*SI Appendix*, Fig. S8*G*). Overall, pseudotime analysis of *Lrig1*^+^ epithelial cells revealed distinct differentiation trajectories toward both stratified squamous and pseudostratified secretory lineages, with many cells occupying early stages of differentiation.

### 1.5. *Lrig1*-Expressing Cells Repopulate Laryngeal and VF Epithelium in Response to Injury.

**Fig. 5.**
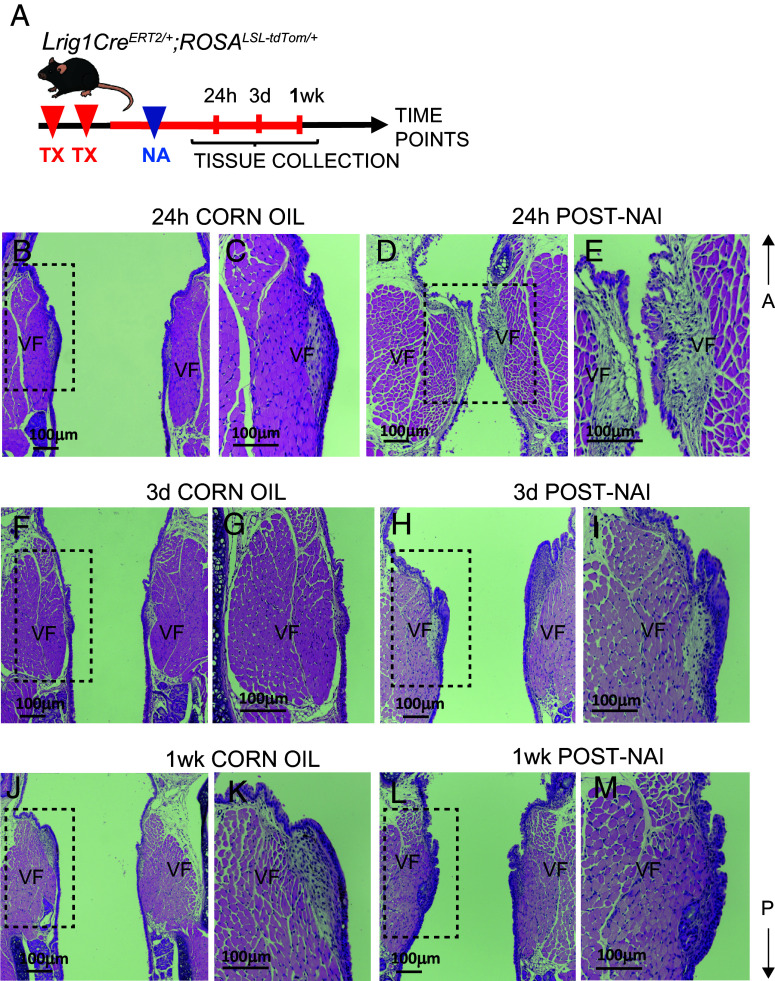
Assessment of morphology following NA injury in *Lrig1^CreERT2/+^*; *ROSA^LSL-tdTom/+^*mice. (*A*) Schematic of experimental model and strategy. (*B*–*M*) H&E coronal sections through mid-membranous VF region in control and NA injected mice 24 h post-NAi (*B*–*E*); 3d post-NAi (*F*–*I*) and 1wk post-NAi (*J*–*M*). Abbreviations: A, anterior; mo, month; NA, naphthalene; NAi, naphthalene injury; P, posterior; TX, tamoxifen; V, ventral; VF, vocal fold; wk, week.

**Fig. 6.**
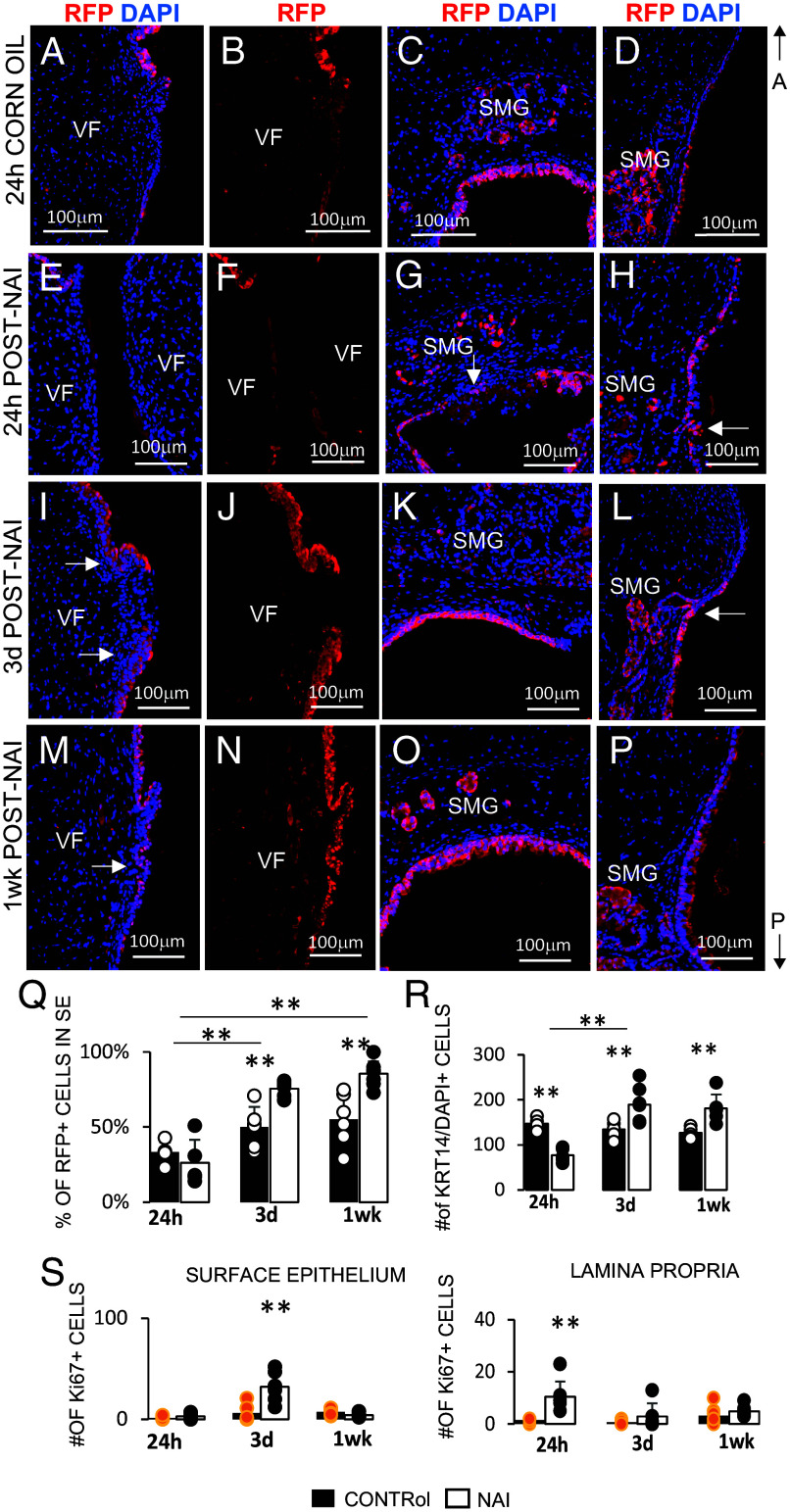
Lineage tracing in *Lrig1^CreERT2/+^*; *ROSA^LSL-tdTom/+^* mice following NA injury. (*A*–*D*) IF for anti-RFP (red) and DAPI in VFs (*A* and *B*), supraglottis (*C*), and subglottis (*D*) in control mice 24 h post corn oil injection. (*E*–*H*) IF for anti-RFP (red) and DAPI in VFs (*E* and *F*), supraglottis (*G*), and subglottis (*H*) in mice 24 h post-NAi. (*I*–*L*) IF for anti-RFP (red) and DAPI in VFs (I and *J*), supraglottis (*K*), and subglottis (*L*) in mice 3d post-NAi. (*M*–*P*) IF for anti-RFP (red) and DAPI in VFs (*M* and *N*), supraglottis (*O*), and subglottis (*P*) in mice 1wk post-NAi. White solid arrows in *G*–*I*, *L*, and *M* denote incorporated RFP+ cell into SE. (*Q*) Percentage of RFP+ cells in SE of VF mid-membranous region across timepoints in control and NA injured mice. (*R*) Number of KRT14+ DAPI cells in SE of VF mid-membranous region across timepoints in control and NA injured mice. (*S*) Number of Ki67 cells in SE and Lamina propria of VF mid-membranous region across timepoints in control and NA injured mice. Data presented were obtained from 3 biological replicates (n = 3). Error bars with individual data points represent ± SEM. One-Way ANOVA of variance for independent or correlated samples statistical analysis along with the Plus Tukey HSD test were used to confirm statistical significance, *P ≤ 0.05 and **P ≤ 0.01 (**). Abbreviations: A, anterior; h, hour; d, day; NAi, naphthalene injury; P, posterior; RFP, red fluorescent protein; SE, surface epithelium; SMG, submucosal glands; VF, vocal fold; wk, week.

To investigate whether *Lrig1*^+^ cells possess progenitor-like properties and are capable of repairing damaged epithelium, we performed naphthalene (NA)-induced injury. Previous studies in the larynx have demonstrated that NA exposure affects the stratified VF epithelium and secretory cells (21). To examine this, we generated *Lrig1Cre^ERT2^;ROSA^LSL-tdTom/+^* reporter mice. These mice received TX via two consecutive injections, followed by a single dose of NA (Fig. 5*A*). Mice injected with corn oil served as controls. Laryngeal tissues were collected at 24 h, 3d, and 1wk post–NA injury (post-NAi) (Fig. 5 *B*–*M*). Our data demonstrate successful induction of epithelial injury (Fig. 5 *B*–*E*). At 24 h post-NAi, VF epithelial cells were visibly damaged and began sloughing off, with cellular debris evident in the lumen compared to controls. By 3d and 1 wk post-NAi, we observed pronounced epithelial folding and expansion Fig. 5 *H*, *I*, *L*, and *M*), relative to uninjured controls (Fig. 5 *F*, *G*, *J*, and *K*). Lineage tracing confirmed that epithelial shedding had occurred in the VF region at 24 h post-NAi, with faint background staining detected in the VF, supraglottis, and subglottis compared to controls (Fig. 6 *A*–*H*). In the epiglottis and subglottis, new *Lrig1*-derived cells began repopulating surface epithelium (SE) (Fig. 6 *G* and *H*). By 3d and 1wk post-NAi, we observed near-complete repopulation of SE with RFP^+^ cells in VFs, supraglottis, and subglottis (Fig. 6 *I*–*P* *SI Appendix*, Fig. S9 *A*–*L*).

We quantified percentage of RFP^+^ cells in the VF SE (Fig. 6*Q*) and found a significant increase from 23% at 24 h post-NAi to 76% at 3d (*P* = 0.000001479) and 86% at 1wk post-NAi (*P* = 0.00000001759), consistent with RFP^+^ cell repopulation observed in histological sections. Additionally, we observed a significant elevation of RFP^+^ cells within the injured VF SE compared with controls at 3d (*P* = 0.008613) and 1wk (*P* = 0.0008522) post-NAi (Fig. 6*Q*), correlating with epithelial expansion in injured tissues. To further address this, we quantified KRT14^+^/DAPI^+^ epithelial cells in VF SE (Fig. 6*R* and *SI Appendix*, Fig. S10 *A*–*F*). At 24 h post-NAi, the number of KRT14^+^/DAPI^+^ cells in injured larynges were significantly reduced compared with controls (*P* = 0.000615), consistent with epithelial sloughing. By contrast, at 3d and 1wk post-NAi, the number of KRT14^+^/DAPI^+^ cells were significantly elevated compared with controls (*P* = 0.004582 and *P* = 0.0008522, respectively), correlating with epithelial folding and expansion. Finally, we assessed whether this epithelial expansion at later recovery stages was associated with increased cell proliferation by quantifying Ki67^+^ cells in VF epithelium and lamina propria (Fig. 6*S*). We found a significant enrichment of Ki67^+^ cells in epithelium at 3d post-NAi, which was preceded by a significant enrichment of Ki67^+^ cells in lamina propria at 24 h post-NAi, as further confirmed by histological staining (*SI Appendix*, Fig. S10 *G*–*L*). Together, these results demonstrate that *Lrig1*^+^ cells contribute to epithelial repair in the larynx/VF following NA-induced injury, with early cell loss followed by expansion and proliferation that restore SE.

### 1.6. *Notch1* Signaling Maintains Quiescence in *Lrig1*^+^ Epithelial Stem/Progenitor Cells.

**Fig. 7.**
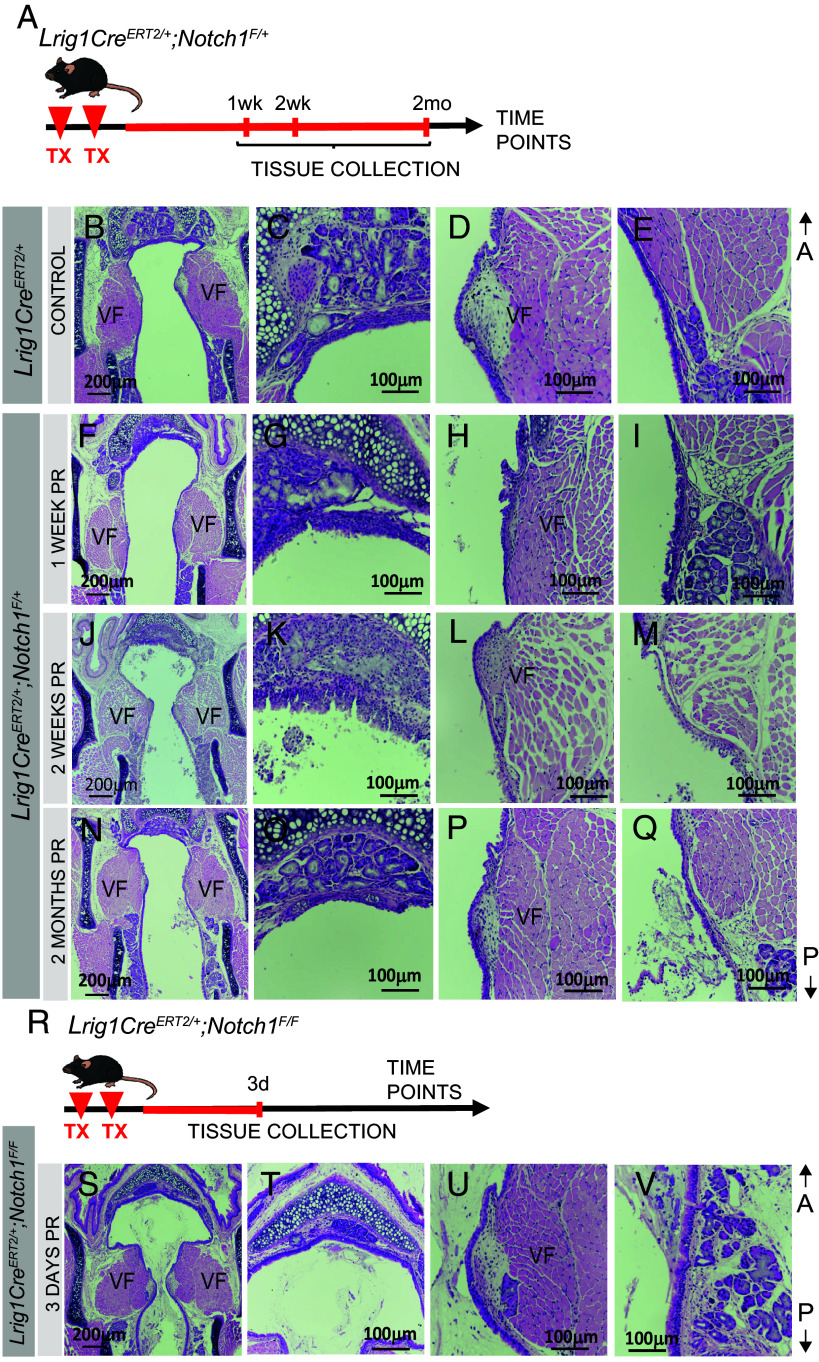
Assessment of morphology following conditional deletion of *Notch1* in *Lrig1^CreERT2/^*^+^ mice. (*A*) Schematic of the experimental model and strategy. Directional axes are indicated. (*B*–*E*) H&E coronal sections through mid-membranous VF region in control mice, showing entire larynx (*B*), supraglottis (*C*), VFs (*D*), and subglottis (*E*). (*F*–*I*) H&E coronal sections through mid-membranous VF region in *Lrig1^CreERT2/+^*; *Notch1^F/+^* heterozygous mice 1-week post-recombination, showing entire larynx (*F*), supraglottis (*G*), VF (*H*), and subglottis (*I*). (*J*–*M*) H&E coronal sections through mid-membranous VF region in *Lrig1^CreERT2/+^*; *Notch1^F/+^* heterozygous mice 2-weeks post-recombination, showing entire larynx (*J*), supraglottis (*K*), VF (*L*), and subglottis (*M*). (*N*–*Q*) H&E coronal sections through mid-membranous VF region in *Lrig1^CreERT2/+^*; *Notch1^F/+^* heterozygous mice 2-wk post-recombination, showing entire larynx (*N*), supraglottis (*O*), VF (*P*), and subglottis (*Q*). (*R*) Schematic of the experimental model. (*S*–*V*) H&E coronal sections through mid-membranous VF region in *Lrig1^CreERT2/+^*; *Notch1^F/F^* homozygote mice 3 d post-recombination, showing entire larynx (*S*), supraglottis (*T*), VF (*U*), and subglottis (*V*). Abbreviations: A, anterior; mo, month; P, posterior; TX, tamoxifen; V, ventral; VF, vocal fold; wk, week.

To investigate potential contribution of *Lrig1+* cells to VF disease, we disrupted *Notch1* signaling, a critical regulator of cell fate determination and differentiation (19). To conditionally delete *Notch1* in *Lrig1*^+^ expressing cells, we generated *Lrig1Cre^ERT2/+^; Notch1^F/+^* and *Lrig1Cre^ERT2/+^; Notch1^F/F^* conditional mutants by mating *Lrig1Cre^ERT2/+^* mice with *Notch1^F/F^* mice. These mice were injected with two TX doses (Fig. 7). *Lrig1Cre^ERT2+/−^* mice were used as controls. Whole larynges were collected at 1wk, 2wk, and 2mo PR in heterozygote animals (Fig. 7*A*). Due to the lethality of extended mouse maintenance in *Lrig1Cre^ERT2/+^; Notch1^F/F^* mutants, tissues were collected only at 3d PR (Fig. 7*R*).

In heterozygous mutants, the most prominent epithelial changes were observed at 2 wk PR (Fig. 7 *B*–*Q*). These changes included uncontrolled epithelial growth leading to epithelial hyperplasia, primarily localized to the supraglottis, accompanied by abundant cellular debris within the lumen. In contrast, both control and 1wk heterozygous larynges showed no notable alterations in laryngeal or VF morphology. By 2mo PR, aberrant epithelium in heterozygotes had largely resolved, although some debris persisted in the lumen. In homozygous mutants, we observed pronounced mucus accumulation and cellular debris in the laryngeal lumen, along with markedly enlarged VFs that significantly narrowed the airway entrance down through to the trachea (Fig. 7 *S*–*V*).

To better characterize epithelial phenotype, we performed IF staining to assess overall epithelial integrity and potential expansion of basal and secretory cells. In control mice, double staining for P63 and KRT14 revealed normal morphology of the basal P63^+^/KRT14^+^ cell layer in the supraglottis, VFs, and subglottic area (*SI Appendix*, Fig. S11 *A*–*F*) and low proliferation activity (*SI Appendix*, Fig. S11*J*). In the mid-membranous VFs specifically, P63^+^ basal cells were arranged in a single layer with relatively weak staining intensity (*SI Appendix*, Fig. S11*D*). Epithelium appeared compact, as evidenced by strong E-cadherin (ECAD) staining (*SI Appendix*, Fig. S11 *G*–*I*). SCGB1A1 expression was observed in the SMGs, as well as in the SE, particularly in the subglottis (*SI Appendix*, Fig. S11 *K*–*N*).

**Fig. 8.**
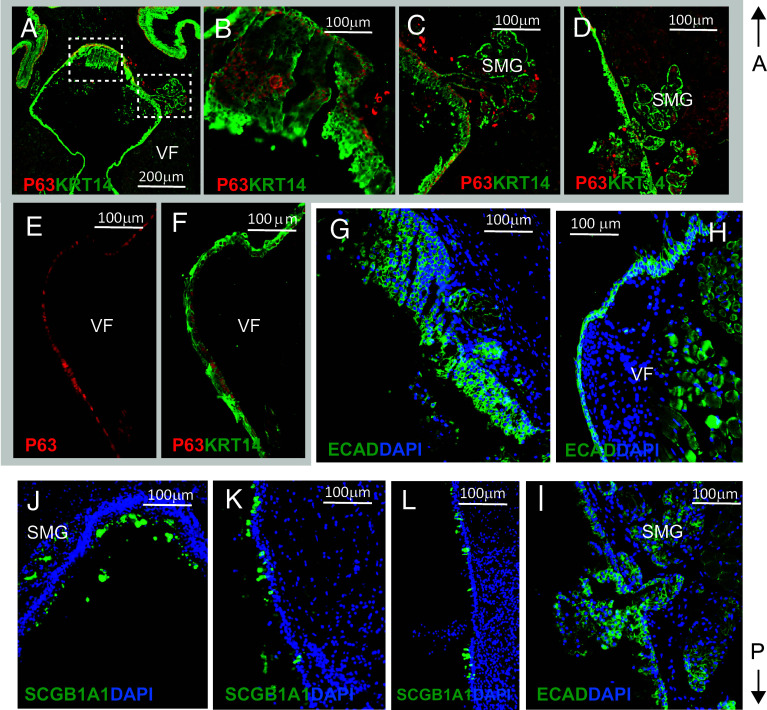
IF for assessment of epithelial integrity and cell type distribution in heterozygous *Lrig1^CreERT2/+^*; *Notch1^F/+^* mice 2 wk PR. (*A*–*F*) Double IF with anti-P63 (red) and anti-KRT14 (green) showing morphology of P63+ basal cell layer in the larynx (*A*), supraglottis (*B* and *C*), subglottic region (*D*), and VFs (*E* and *F*). Bracketed regions in panel *A* are shown at higher magnification in panels *B* and *C*, respectively. (*G*–*I*) IF staining with anti-E-cadherin (ECAD, green) and DAPI (blue) showing disruption of epithelial adherens junctions in supraglottis (*G*), VFs (*H*), and subglottis (*I*). (*J*–*L*) IF staining with anti-SCGB1A1 (green) and DAPI (blue) showing the distribution of secretory cells within the SE and SMGs in the supraglottis (*J*), VFs (*K*), and subglottic region (*L*). Abbreviations: SE, surface epithelium; SMG, submucosal glands; VF, vocal fold.

**Fig. 9.**
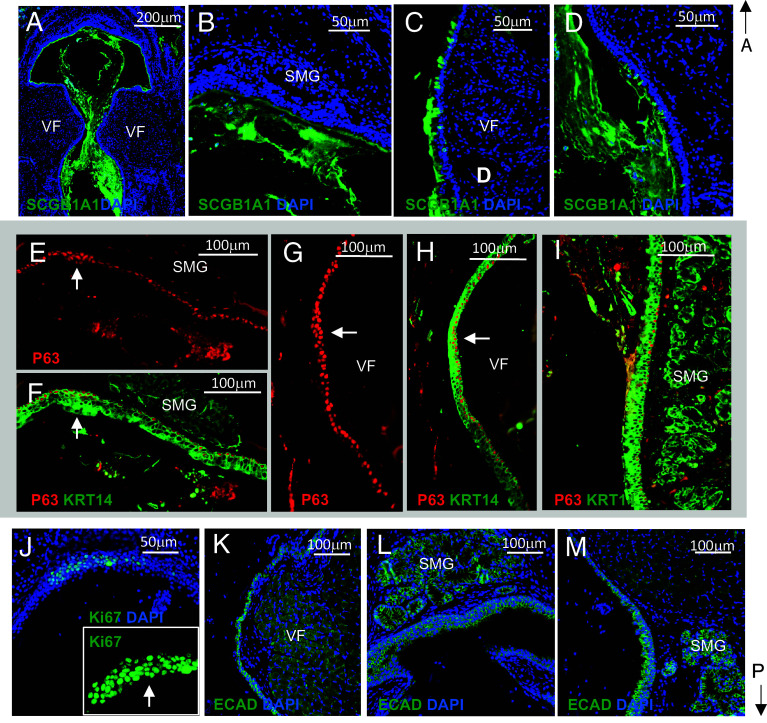
IF for assessment of epithelial integrity and cell type distribution in homozygous *Lrig1^CreERT2/+^*; *Notch1^F/F^* mice 3 d PR. (*A*–*D*) IF with anti-SCGB1A1 (green) and DAPI (blue) showing massive expansion of secretory cells and massive accumulation of mucus and cell debris in laryngeal lumen (*A*), supraglottis (*B*), VFs (*C*), and subglottic region (*D*). (*E*–*I*) Double IF with anti-P63 (red) and anti-KRT14 (green) showing morphology of P63+ basal cell layer in the supraglottis (*E* and *F*), VFs (*G* and *H*), and subglottic region (*I*). White solid arrows in *E*, *F*, *G*, and H denote P63 cell expansion. (*J*) Anti-Ki67 IF staining (green) and DAPI in supraglottis. The white solid arrow denotes Ki67 cell expansion. (*K*–*M*) IF staining with anti-E-cadherin (ECAD, green) and DAPI (blue) showing compactness of the epithelium in VFs (*J*), supraglottis (*K*), and subglottis (*L*). Abbreviations: SMG, submucosal glands; VF, vocal fold.

In heterozygous mice at 1wk PR, we observed early signs of epithelial thickening and P63+ cell expansion in the supraglottic and subglottic areas, accompanied by increased cell proliferation (*SI Appendix*, Fig. S12 *A*–*G* and *J*). P63^+^ basal cells lining the VFs remained organized in a single layer, but their expression intensity was noticeably stronger (*SI Appendix*, Fig. S12*D*) compared to controls (*SI Appendix*, Fig. S11*D*). E-cadherin (ECAD) staining revealed compact epithelium (*SI Appendix*, Fig. S12 *H* and *I*). Anti-SCGB1A1 staining showed positive staining in SMGs and SE (*SI Appendix*, Fig. S12 *K*–*N*). In heterozygous mutants at 2wk PR, IF revealed random and excessive accumulation of P63^+^ cells within KRT14^+^ epithelium (Fig. 8 *A*–*F*), particularly in the supraglottis. The expansion of P63+ cells was accompanied by compromised cell junctions (Fig. 8 *G*–*I*), leading to formation of noticeable gaps that facilitated epithelial sloughing. Additionally, SMGs were enlarged and incorporated into SE, contributing to increased mucus production and debris accumulation (Fig. 8 *C*, *D*, and *I*). In mid-membranous VFs, P63^+^ basal cells remained organized in a single layer, but their expression was noticeably stronger, and cells appeared more tightly packed (Fig. 8*E*) compared to controls (*SI Appendix*, Fig. S11*D*). We observed a marked expansion of SCGB1A1^+^ secretory cells in luminal SE lining the supraglottis (Fig. 8 *J*–*L*). In homozygous animals, IF confirmed massive mucus accumulation and marked expansion of SCGB1A1^+^ secretory cells (Fig. 9 *A*–*I*). We observed P63+ cell expansion in the supraglottis and mid-membranous VFs (Fig. 9 *E*–*H*), accompanied by increased proliferation (Fig. 9*J*). Epithelium appeared more compact as compared to heterozygotes (Fig. 9 *K*–*M*). In conclusion, our study demonstrates that disruption of *Notch1* signaling in *Lrig1*^+^ cells significantly impairs laryngeal/VF tissue homeostasis, affecting epithelial thickness, adherens junctions, mucus production, and compromising airway patency.

## 2. Discussion

We have significantly advanced understanding of candidate laryngeal and VF stem cells by defining their identity, characterizing their properties, and investigating their potential contributions to tissue repair and aberrant mucosal remodeling. Our findings support our hypothesis that *Lrig1* marks quiescent cells that contribute to long-term laryngeal and VF homeostasis and tissue repair. Through a combination of lineage tracing and scRNA-seq, we identified multiple distinct *Lrig1*-expressing populations. These populations overlapped with *Lrig1*-derived (*Lrig1*^−^) cells, highlighting significant heterogeneity and potential functional specialization within laryngeal and VF tissues. Each major cell type contains a subset of quiescent stem cells that preferentially contribute to tissue renewal and repair in response to physiological demands or injury (*SI Appendix*, Fig. S13). Importantly, analogous LRIG1^+^ populations were detected in the human larynx and VF mucosa, underscoring their potential relevance to human VF biology and disease.

Our findings revealed that *Lrig1*^+^ cell populations are enriched among less differentiated epithelial cell types, including basal, parabasal, and immature secretory (club) cells within SE and SMGs. These populations exhibit both transcriptional flexibility and protective spatial localization. They are strategically positioned within niches that are shielded from direct environmental and mechanical stressors, creating a microenvironment that supports cellular quiescence and a low proliferative state. In stratified epithelia, basal and parabasal layers lie beneath the luminal surface, protecting resident stem cells from mechanical abrasion, pathogens, and inflammatory insults (22) (*SI Appendix*, Fig. S13). This protected location may facilitate rapid response upon tissue injury, as these cells can proliferate and replenish the stem cell pool. Similarly, in pseudostratified epithelia, immature club cells have been shown to serve as facultative progenitors, capable of transitioning from a quiescent to an active state in response to injury or physiological demand (8, 23).

In addition to the spatial distribution of *Lrig1^+^* cells, we demonstrated that *Lrig1*^+^ cells—particularly the *Lrig1^High^* group—exhibited a transcriptional profile distinct from *Lrig1^−^* and *Lrig1^Low^* cells, characteristic of a reversible cell cycle arrest (G_0_ phase) (4). This state is defined by suppression of cell type–specific differentiation genes and a concurrent upregulation of genes involved in RNA metabolism, epigenetic regulation and protein ubiquitination. Such a profile supports stem cell function, longevity, and transcriptional flexibility without committing to differentiation (23). As reported elsewhere, quiescent stem cells are known to express a repertoire of RNA-binding proteins and regulatory noncoding RNAs, which play crucial roles in preserving stemness and fine-tuning gene expression (24). Similarly, epigenetic regulation plays a pivotal role in maintaining stem cell identity, while protein ubiquitination functions as a crucial mechanism of protein quality control in stem cells, safeguarding stem cell function, survival, and proper differentiation (25, 26). Conversely, *Lrig1^−^* and *Lrig1^Low^* cells prioritized expression of lineage-specific effector genes, reflecting a shift toward terminal differentiation. These cells also exhibited a higher proliferation rate, supporting their active role in tissue turnover and maturation (16). Consequently, their transcriptome is narrower and enriched for terminal differentiation markers.

### 2.1. *Lrig1* Expression in Epithelial Cell Populations.

Our results align with the literature, supporting the role of *Lrig1* as a marker of quiescent stem cells in epithelial cell populations. In the esophagus, which shares similar SSE with VFs, *Lrig1* marks a subset of basal cells scattered throughout the epithelium that contribute to esophageal growth and homeostasis (27). Following radiation injury, *Lrig1*-positive cells persist and expand to repopulate the damaged epithelium. Similarly, in oral SSE, *Lrig1*-enriched basal cells are located in regions with low rates of cell proliferation and enter the cell cycle during wound healing or in response to masticatory stress (13). Last, in the airway epithelium, *Lrig1* is heterogeneously expressed during homeostasis and labels a subpopulation of basal cells with enhanced proliferative and self-renewal capacity in mice and humans (28, 29).

### 2.2. *Lrig1* Expression in Nonepithelial Cell Populations.

We identified *Lrig1*-expressing cells in nonepithelial populations, suggesting potential roles beyond its established function in epithelial stem cell regulation. Only one study has reported *Lrig1* expression in mesenchymal compartments: Seidel et al. (30) identified *Lrig1* as a marker of both epithelial and mesenchymal stem cells in the adult mouse incisor. The presence of *Lrig1*^+^ mesenchymal cells in both mechanically active tissues raise possibility that similar progenitor populations in the larynx contribute to lamina propria maintenance and regeneration under mechanical stress imposed by VF vibration. Our data revealed *Lrig1* expression in EC, suggesting a previously underappreciated role for *Lrig1* progenitor cells in vascularization. Though not traditionally linked to endothelial biology, *Lrig1* has been implicated in angiogenesis and vascular remodeling in other tissues (31). *Lrig1^+^* presence in EC in VF mucosa indicates possible involvement in vascular homeostasis or repair, especially during inflammation or injury. Neovascularization plays a key role in VF pathologies, particularly those involving chronic inflammation, mechanical stress, or neoplastic transformation. Some VF lesions show increased capillary density, vascular remodeling, and upregulation of *VEGF* and *bFGF*, promoting inflammation and ECM remodeling (32).

### 2.3. Role of *Lrig1*^+^ Cells in Epithelial Repair Following Injury.

We have demonstrated that *Lrig1*^+^ cells play an important role in epithelial repopulation in response to injury. To specifically evaluate epithelial repair, we employed NA injection, a well-established model that selectively ablates Clara/club-like epithelial cells through cytochrome P450–mediated metabolism of NA, thereby allowing the assessment of stem cell contributions to epithelial regeneration (33). Our data suggest that the source of the *Lrig1*^+^ progenitor pool is not limited to SE but may also include SMGs. This finding is consistent with previous studies in trachea and other airway regions, which demonstrate that SMGs serve as a reservoir of progenitor cells contributing to epithelial regeneration following damage (34).

### 2.4. Disruption of Quiescence.

Regulation of stem cell quiescence is crucial for maintaining tissue homeostasis and ensuring proper stem cell function over time. Proper regulation of quiescence prevents premature differentiation or excessive proliferation, both of which could compromise tissue integrity or lead to tumorigenesis and/or stem cell depletion. In this study, we used conditional ablation of *Notch1* in *Lrig1*-expressing cells to drive them out of quiescence. Our scRNA-seq data revealed that *Notch1* was predominantly active in BEC, SBEC, and SEC. Upon *Notch1* inactivation, we observed significant changes in epithelial compartments, including hyperplasia, disruption of cell junctions, and expansion of secretory cells. Homozygous mutants exhibited most severe phenotypes, including mucus accumulation that obstructed the airway, which likely contributed to lethality.

Aberrant distribution or increased mucus production and hyperplastic epithelial changes are common concerns in laryngology, often associated with chronic inflammatory conditions such as chronic laryngitis and VF benign lesions (11). In these contexts, epithelial remodeling, mucus hyperproduction, and impaired clearance of mucus can lead to symptoms like throat clearing, chronic cough, voice changes, and airway obstruction (10). Pathologically, this can reflect hyperplasia of mucus-secreting cells or SMG enlargement. In SSE, genetic ablation of *Notch1* in adult basal cells of mouse epidermis and cornea leads to epithelial thickening, deregulated squamous cell differentiation, hyperkeratosis, dermal inflammation, and eosinophilic infiltrates, all of which disrupt tissue homeostasis, barrier function, and promote inflammatory responses (14, 35). Similarly, in human airway epithelium, activation of *Notch* receptors shifts basal epithelial cells toward a secretory lineage, disrupting the normal balance of cell types and potentially impairing barrier defense mechanisms (36). Besides genetic ablation, *Notch1* can be regulated by mechanical forces and/or inflammation. In EC, *Notch1* functions as a mechanosensor, integrating laminar shear stress cues to maintain junctional integrity, suppress proliferation, and regulate intracellular calcium signaling (37). Given the high mechanical demand of the VFs, this suggests a potential role for *Notch1* in mediating the relationship between mechanical forces and epithelial/mucosal homeostasis with implications for understanding stress-induced VF tissue remodeling.

### 2.5. Conclusions.

In conclusion, our study identifies *Lrig1^+^* cells and *Notch* signaling as key regulators of VF mucosal homeostasis and secretion. *Lrig1^+^*cells serve as quiescent stem cells supporting long-term tissue repair, with *Notch* signaling guiding their activity. Disruption of this axis leads to epithelial changes and mucus imbalance. These findings lay the groundwork for future strategies to modulate stem cell quiescence and explore alternative activation pathways—such as mechanical stress and inflammation, with broad relevance to regenerative medicine.

## 3. Materials and Methods

Detailed descriptions mouse models, tissue harvesting, generation of transgenic and mutant mouse models, naphthalene treatment, histology and immunofluorence, cell count quantification, single cell dissociation, flow cytometry, single cell library construction and sequencing, sequencing, preprocessing, data analysis, classification and assessment of *Lrig*^+^ and *Lrig1*^−^ and *Lrig1*^+^ cell populations, differential expression analysis with cell type annotation and trajectory inferences are provided in *SI Appendix*.

## Supplementary Material

Appendix 01 (PDF)

## Data Availability

Gene expression data have been deposited in NCBI Geo (GSE298629) (38).
