# Supplementary material for: Lrig1-expressing quiescent stem cells maintain vocal fold mucosal homeostasis via Notch signaling
Source: Proc Natl Acad Sci U S A. 2025 Nov 25;122(48):e2513590122. doi: 10.1073/pnas.2513590122 (PMC12685045; doi:10.1073/pnas.2513590122)
Supplement: Supplementary file 1 — Appendix 01 (PDF) [file pnas.2513590122.sapp.pdf]

Material and Methods

Mouse Models and Tissue Harvesting.
Lrig1Cre^{ERT2} (JAX # 018418), *Notch1*$^{tm2Rko/GridJ}$ (*Notch1*$^{LoxP/loxP}$ also known as *Notch1*$^{F/F}$) (JAX # 006951), and *ROSA26*$^{CAG\text{-}loxp\text{-}stop\text{-}loxp\text{-}tdTomato}$ (JAX #007909) were used. Data reported herein have been compiled from, *Lrig1Cre*$^{ERT2/+}$, heterozygous, and conditional knockout animals.

Generation of Transgenic and Mutant Mouse Models.
Lrig1Cre$^{ERT2/+}$ mice were maintained by crossing female heterozygotes *Lrig1*$^{CreERT2/+}$ with WT male littermates on a C57BL/6 J background. *Lrig1*$^{CreERT2/+}$ mice exhibited normal phenotypes and were identical to WT colony controls (21). *Lrig1*$^{CreERT2/+}$ females were crossed to *ROSA26*$^{CAG-LSL-tdTom/tdTom}$ males to generate *Lrig1*$^{CreERT2/+}$*; ROSA26*$^{CAG-LSL-tdTomato/+}$ (here referred to as *ROSA*$^{LSL-tdTom/+}$) reporter mice. *Lrig1*$^{CreERT2/+}$*; ROSA26*$^{LSL-tdTom/+}$ without TX injection and *ROSA*$^{LSL\text{-}tdTom/+}$ were used as controls to establish the baseline *tdTom* levels for lineage tracing experiments and FASC. *Lrig1*$^{CreERT2/+}$ females were mated with *Notch1*$^{F/F}$ males to generate *Lrig1Cre*$^{ERT2/+;\ Notch1F/+}$ heterozygotes. *Lrig1*$^{CreERT2/+}$*; Notch1*$^{F/+}$ were crossed to *Notch1*$^{F/F}$ to generate conditional homozygote mutants *Lrig1*$^{CreERT2/+}$*; Notch1*$^{F/F}$. *Lrig1*$^{CreERT2/+}$ mice were used as controls for these experiments.

Naphthalene Treatment.
Mice received 0.275 mg/per gram body weight of Naphthalene (Millipore Sigma) interperitoneally.

Histology and Immunofluorescence.
Mice and human larynges were fixed in 4% paraformaldehyde and processed for immunostaining. Primary antibodies were incubated overnight at 4 °C, followed by secondary antibodies for 1.5 h at room temperature RT). Slides were mounted with DAPI and stored at 4 °C. Imaging was performed using a Nikon Eclipse Ti2 inverted microscope with DS-Ri2 camera and NIS Elements software (v5.21.01).

Cell Count Quantification.
Total DAPI+ epithelial cells, as well as RFP+, KRT14+, and Ki67+ labeled cells were manually counted using ImageJ. For RFP^{+}, we calculated the percentage of RFP^{+} cells, normalized to total DAPI^{+} epithelial cells, for comparisons at 24 h, 1 d, 3 d, 1 wk, 2 wks, and 3 mo. For the injury model, we analyzed sections at 24 h, 3 d, and 1 wk post-NAi.

Statistical Analysis.
One-way ANOVA was used to compare group means for percentage of RFP+ as well as relative abundance of Ki67+ and KRT14/DAPI with Tukey's HSD post hoc test to determine pairwise comparisons. Results are reported as mean ± SD and were considered statistically significant at $^{*}P \leq 0.05$ or $^{**}P \leq 0.01$.

Single Cell Dissociation and Flow Cytometry Cell Sorting.
Larynges were collected from *Lrig1*$^{CreERT2/+}$*; ROSA26*$^{LSL-tdTom/+}$ mice, WT controls, and *ROSA26*$^{LSL-tdTom/+}$ mice. Tissues were minced, dissociated, and pooled. After centrifugation, supernatants were discarded and cells filtered through a strainer to remove aggregates. Pellets were resuspended in ACK lysing buffer to eliminate erythrocytes. For flow cytometry, cells were washed, centrifuged, resuspended, and passed through a strainer before sorting on a FACSAria2. WT and *ROSA26*$^{LSL-tdTom/+}$ cells served as negative and background controls. FACS data were analyzed using J Flow software.

Single-Cell RNA-Seq Library Construction and Sequencing.
scRNA-seq libraries were prepared using Chromium GEM-X 3′ v4 Reagent Kit (10x Genomics). Reverse transcription and barcoding were followed by cDNA amplification and purification with SPRIselect beads, quantified via Bioanalyzer 2100. Libraries were sequenced on an Illumina NovaSeq X + using 150 bp paired-end reads.

Preprocessing of 10x scRNAseq and Data Analysis.
Raw reads from 10x single-cell RNA-seq were aligned to the mouse genome (GRCm39) using CellRanger v8.0.1. Low-quality cells were filtered based on >8% mitochondrial content, <500 or >8,000 UMI counts, or <1,000 or >40,000 detected genes. Doublets were removed using DoubletFinder v2.0.4. Batch effects across three biological replicates were well controlled. Data were normalized, scaled, and variable genes selected using Seurat v5.1.0, with log-normalization performed via NormalizeData (scale factor = 10,000).

Classification of Cells Based on the *Lrig1* Gene.
Cells were grouped by normalized *Lrig1* expression: zero-expression cells were labeled *Lrig1*$^{-}$, and those with any expression were *Lrig1*$^{+}$. For further analysis, cells were classified as *Lrig1*^{High} or *Lrig1*^{Low}. All *Lrig1*$^{-}$ cells were assigned to *Lrig1*^{Low}. Among *Lrig1*$^{+}$ cells, those above the sample-specific median were *Lrig1*^{High}; those at or below were *Lrig1*^{Low}.

Assessment of *Lrig1*$^{-}$ and *Lrig1*$^{+}$ Cell Populations.
PCA was performed using Seurat's RunPCA, and top 10 components were used to generate UMAP embeddings. Gene expression was aggregated into pseudobulk matrices at sample and group levels. Differential expression between *Lrig1*$^{-}$ and *Lrig1*$^{+}$ groups was analyzed using *Lrig1* DESeq2 (v1.44.0). DE results were mapped to Entrez IDs via org.Mm.eg.db (v3.19.1) and used for gene set enrichment analysis (GSEA) with clusterProfiler (v4.12.6) using the gseGO function and GO Biological Process ontology.

Dimensionality Reduction and Clustering.
Dimensionality reduction and clustering for *Lrig1*$^{-}$ and *Lrig1*$^{+}$ groups were performed using Monocle3 (v1.3.7). Data subsets were processed into separate cds objects, and PCA was run with preprocess_cds(norm_method = "none", num_dim = 15). UMAP embeddings were generated via reduce_dimension(umap.n _ neighbors = 15, umap.min_dist = 0.1). Clustering was performed using t Leiden algorithm (cluster_cells) on a k-nearest neighbor graph (k = 15), with clusters identified by optimizing partition modularity.

Differential Expression Analysis.
Differential expression analysis was performed using Seurat's FindAllMarkers with the Wilcoxon rank-sum test and Bonferroni correction. Genes expressed in ≥10% of cells and absolute average $\log_2$ fold change >0.25 were considered differentially expressed.

Cell Type Annotation and Cell Proportion.
Manual cell type annotation was performed using cluster-specific marker genes. For each cluster, top 100 upregulated genes (ranked by adjusted *P*-value and $\log_2$ fold change) were matched to known cell-type profiles via CellKb (v2.10). Annotated cell type proportions were calculated within *Lrig1*$^{-}$ and *Lrig1*$^{+}$ groups for downstream comparison.

Trajectory Inference and Pseudotime Analysis.
We used Monocle3 for cell trajectory analysis to infer lineage relationships among *Lrig1*$^{+}$ clusters.

Supplementary materials: *Lungova et al.*

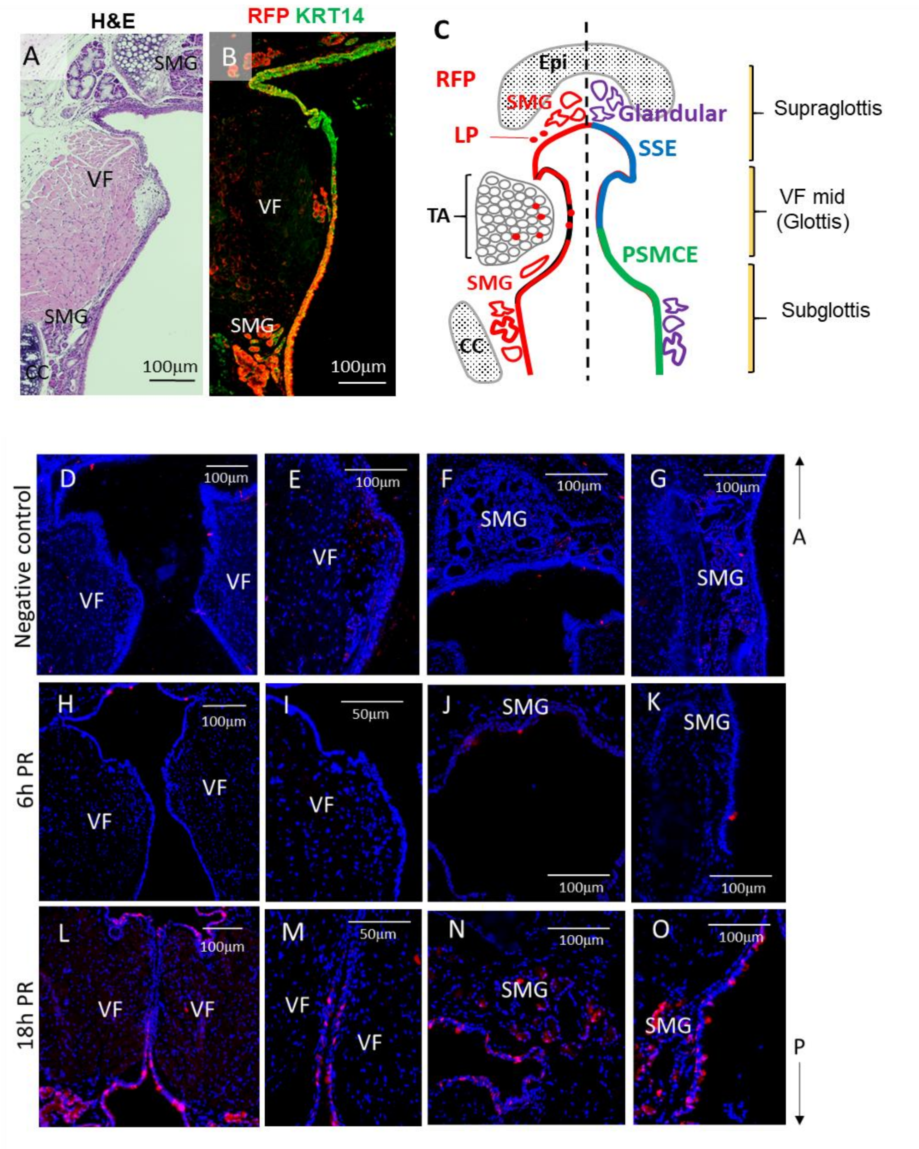

Supplementary Figure 1: Early-Stage Lineage tracing in *tdTomato* red fluorescent reporter in *Lrig1*$^{CreERT2/+}$ mice. (A, B) H&E staining and IF staining with anti-RFP and anti-KRT14 in the coronal mid-membranous VF sections in *Lrig1Cre*$^{ERT2/+;}$*ROSA*$^{tdTom/+}$ mice, showing tissue morphology and co-localization of RFP+ and KRT14 in SMGs and SE. **(C)** Schematic illustration summarizing RFP+ cell distribution and epithelial organization in the coronal mid-membranous section of the larynx. Left panel depicts the distribution of RFP+ cells in SE, SMGs, with some sporadic staining seen in lamina propria, and TA muscle. Right panel provides regional orientation through the larynx, highlighting corresponding epithelial types in each region. **(D-G)** Assessment of the VF Region (D, E), the supraglottis (F), and the subglottis (G) in negative control mice without tamoxifen injection. **(H-K)** Assessment of the VF Region (H,I), the supraglottis (J), and the subglottis (K) in mice 6h post recombination. (L-O) Assessment of the VF Region (L,M), the supraglottis (N), and the subglottis (O) in mice 18h post recombination. Histological analyses were performed in two biological replicates (n=2). Abbreviations: A, anterior; h; hour; CC, cricoid cartilage; Epi; epiglottis; LP, lamina propria; PSMCE, pseudostratified mucociliary epithelium; SMG,

submucosal glands; SSE, stratified squamous epithelium; VF, vocal folds; P, posterior; PR, post-recombination; TA, thyroarytenoid muscle.

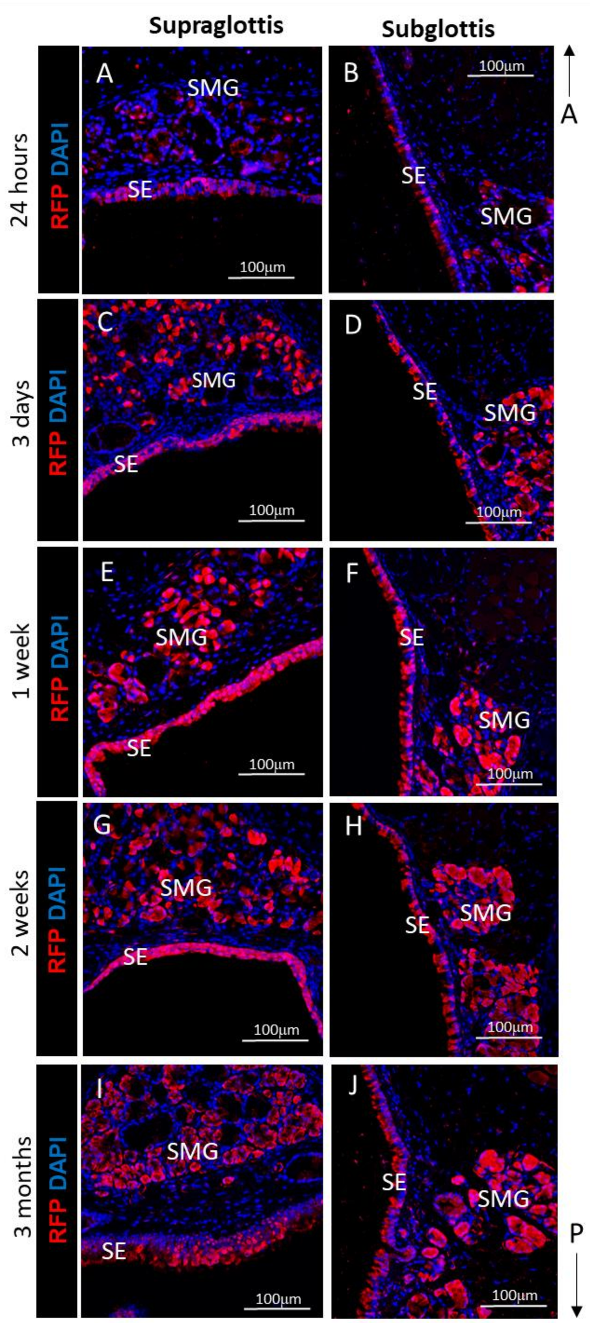

Supplementary Figure 2: Lineage tracing in *tdTomato* red fluorescent reporter in *Lrig1*$^{CreERT2/+}$ mice in supraglottic and subglottic regions. (A, B) Assessment of *tdTom* expressing *Lrig1* cells in supraglotiic and subglottic regions 24h PR; (C, D) 3d PR; (E, F) 1wk PR; (G, H) 2wk PR; and (I, J) 3 mo PR. Directional axes are provided. Histological analyses were performed in three biological replicates (n=3). Abbreviations: A, anterior; SE, surface epithelium; SMG, submucosal glands; VF, vocal folds; P, posterior; PR, post-recombination.

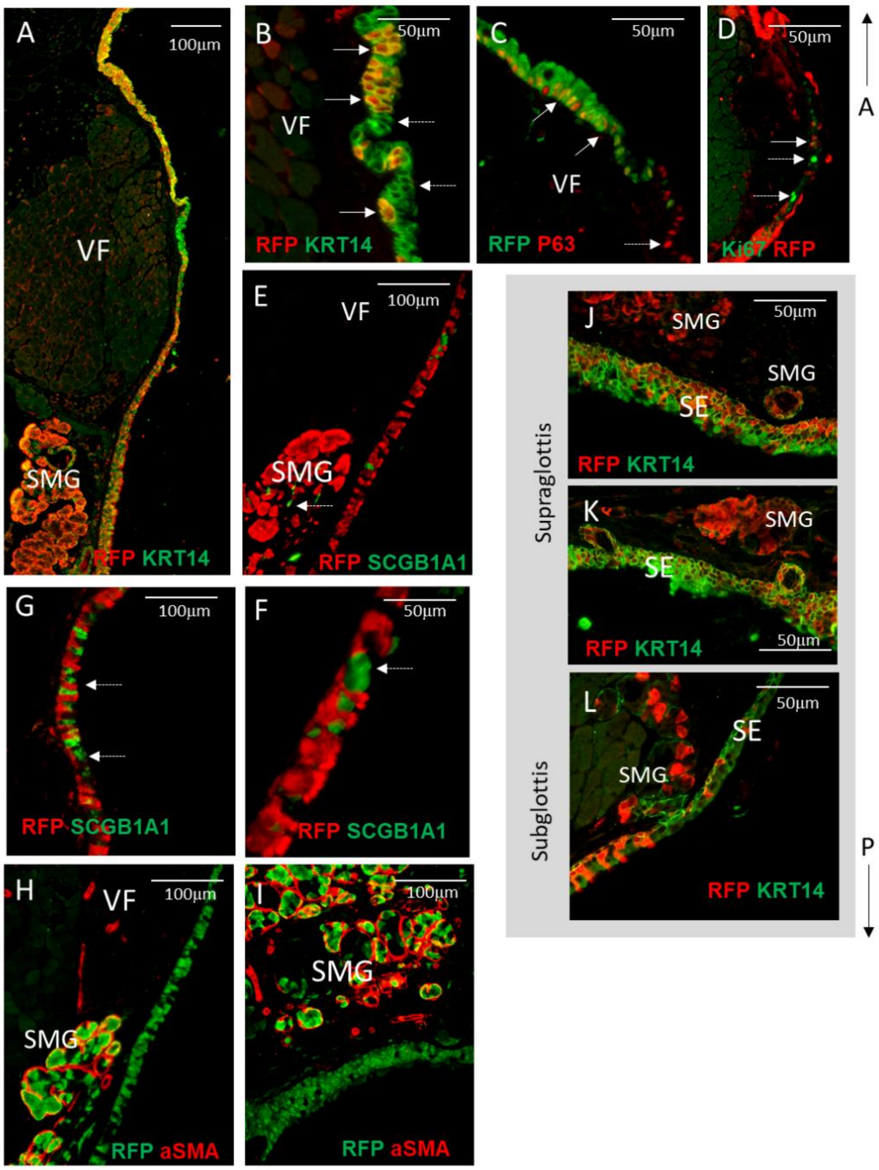

Supplementary Figure 3: Assessment of cell populations containing RFP+ cells. **(A, B)** Co-localization of RFP (red) with cytokeratin KRT14 (green), indicating that RFP+ cells were primarily of epithelial origin. White solid arrows denote RFP+ cells in the basal and suprabasal cell layers. White dashed arrows denote RFP- KRT14+ cells. **(C)** Co-localization of RFP (red) with P63+ cells (green) along the superior margin of the VFs, indicated by solid white arrows. **(D)** Co-localization of RFP (red) with Ki67+ cells (green) in the VFs. White solid arrows denote RFP+ Ki67+ cells. White dashed arrows denote RFP- Ki67+ cells. **(E-G)** Co-localization of RFP (red) with SCGB1A1 cells (green) in the subglottis. White dashed arrows denote lack of RFP signal in SCGB1A1+ cells. **(H,I)** Co-localization of RFP (green) with aSMA myoepithelial cells (red) in submucosal glands in the supraglottis (H) and subglottis (I). **(J-L))** Double immunofluorescent staining for anti-RFP and anti-KRT14 showing interaction between SMGs and the SE in the supraglottic (J, K) and subglottic regions (L). All histological analyses were performed on three biological replicates (n=3). Abbreviations: SE, surface epithelium; SMG, submucosal glands; VF, vocal folds.

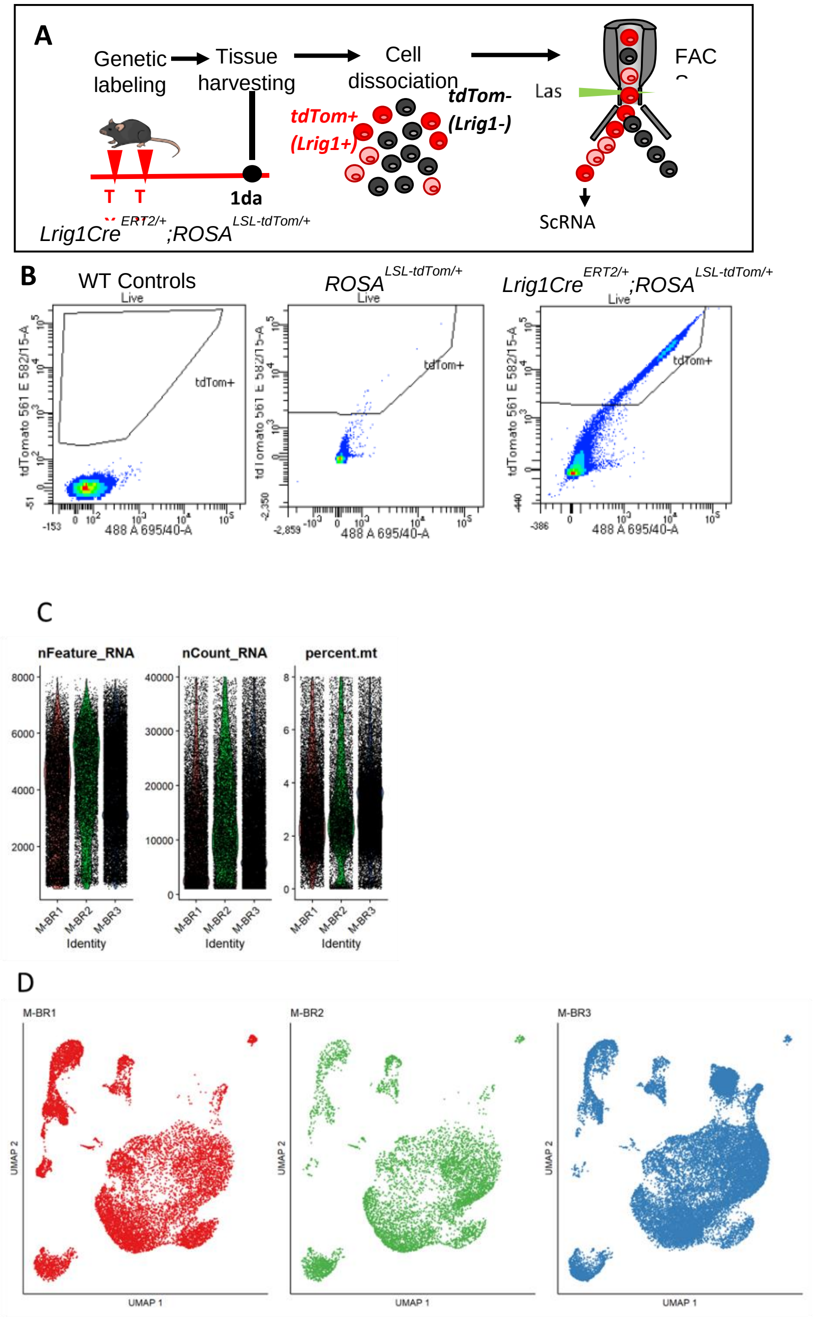

Supplementary Figure 4: Single cell RNA sequencing strategy. (A) Schematic illustration of the workflow for harvesting murine laryngeal tissue, isolating *tdTomato*$^+$ *(tdTom*$^+$*)* cells, followed by enzymatic dissociation and flow cytometrymedicated cell sorting. **(B)** Flow cytometry gating strategy for sorting *tdTom*$^+$ cells, including negative controls: wild-type (WT) and background signal control from *ROSA*$^{LSL-tdTom/+}$ mice. (**C**) Quality control and batch assessment. Violin plots showing quality control summaries for each group: number of detected genes per cell (nFeature_RNA), total UMI counts (nCount_RNA), and percentage of mitochondrial reads (percent.mt). (**D**) UMAP projections of each group (M-BR1, M-BR2, M-BR3) showing similar cell distributions across groups.

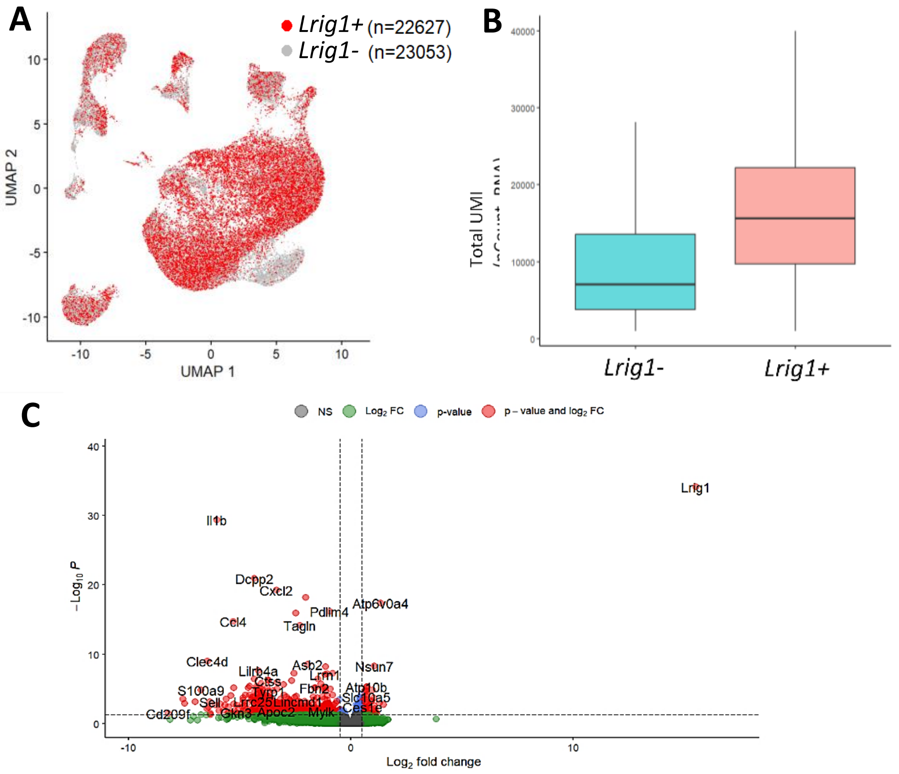

Supplementary Figure 5: Single cell RNA sequencing of *Lrig1*+ and *Lrig1*- cell populations. (A) UMAP visualization of ovelapping *Lrig1*$^{+}$ (red) and *Lrig1*$^{-}$ (grey) cell polutations with the corresponding cell counts. **(B)** Total UMI (nCount_RNA) for *Lrig1*$^{+}$ and *Lrig1*$^{-}$ cell populations. **(C)** Pseudobulk differentially expressed gene analysis in *Lrig1*$^{+}$ amd *Lrig1*$^{-}$ cell populations, Enhanced Volcano graph.

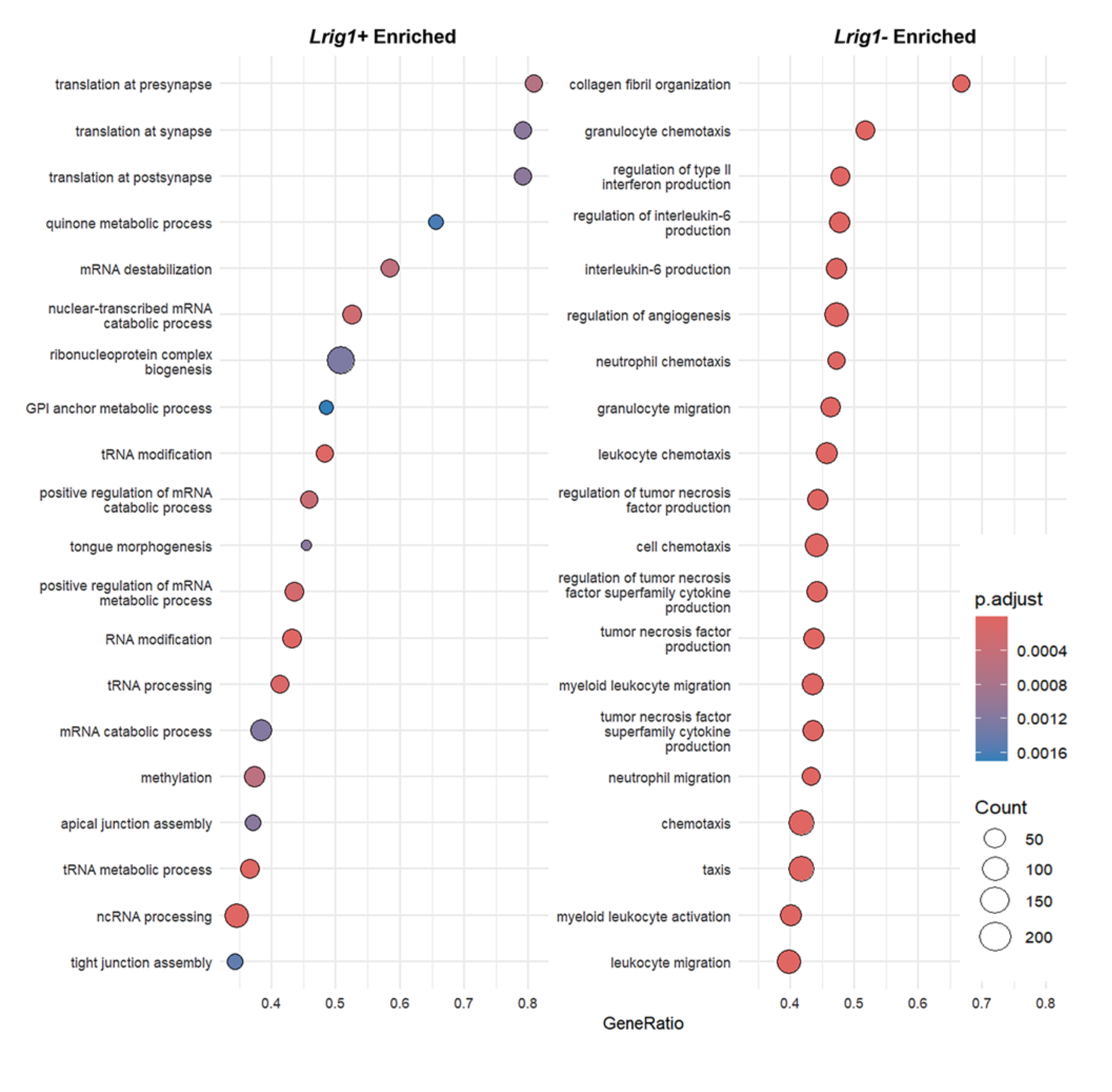

Supplementary Figure 6: Gene set enrichment analysis of *Lrig1*$^{+}$ and *Lrig1*$^{-}$ cell populations. Dot plot of top enriched GO biological processes based on genes ranked by test statistic from the pseudobulk comparison. Pathways enriched toward *Lrig1*$^{+}$ (left) relate to RNA activity. Pathways enriched toward *Lrig1*$^{-}$ (right) involve immune responses and chemotaxis. Dot size indicates gene set size, color reflects adjusted *P*-value, and the x-axis shows the gene ratio.

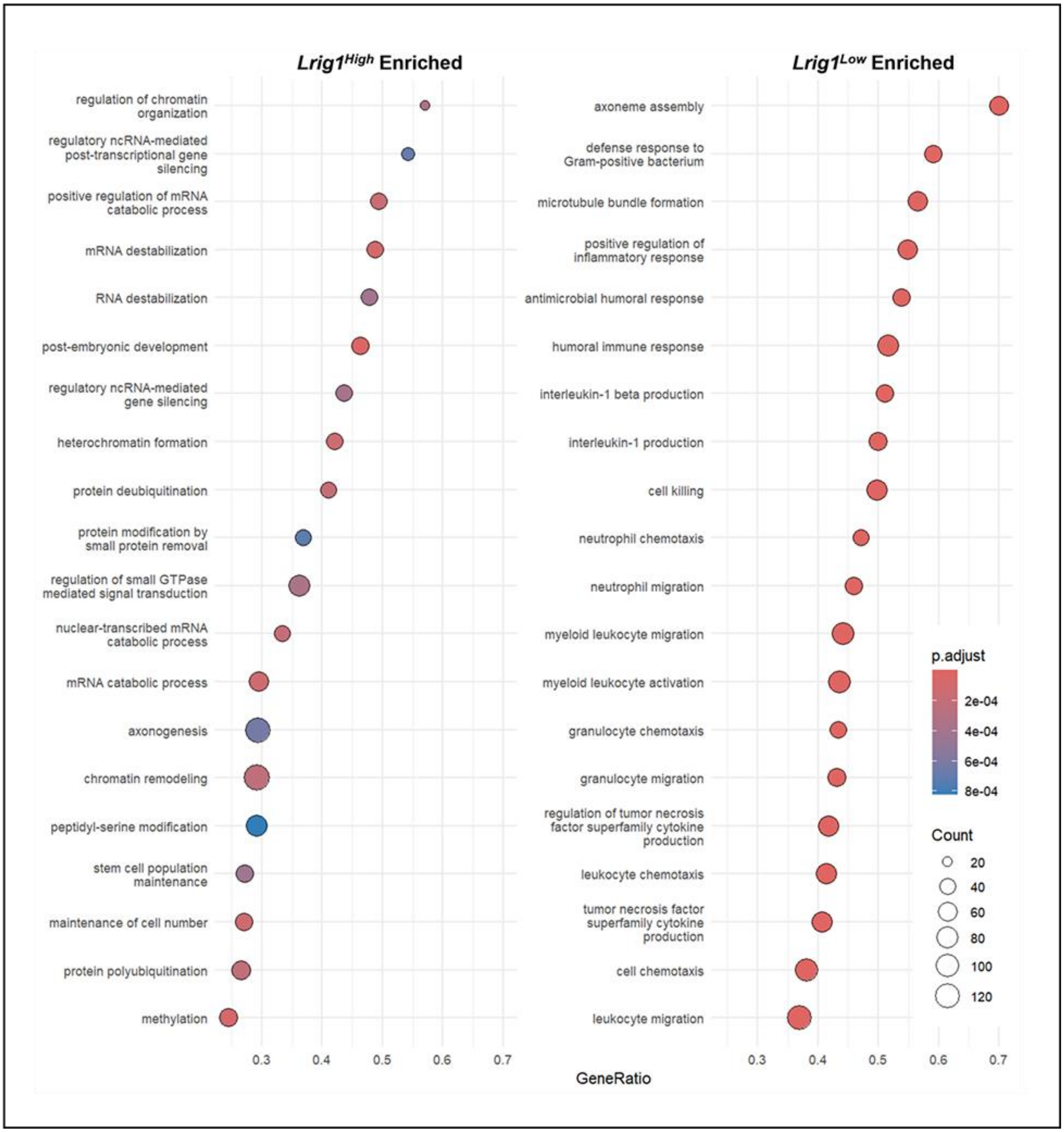

Supplementary Figure 7: Gene set enrichment analysis of *Lrig1*^{High} and *Lrig1*^{Low} cell populations. Dot plot showing the top enriched GO biological processes based on genes ranked by test statistic from the pseudobulk comparison. Pathways enriched toward *Lrig1*^{High} (left) relate to RNA activity and protein modifications. Pathways enriched toward *Lrig1*^{Low} (right) involve immune responses and chemotaxis. Dot size indicates gene set size, color reflects adjusted *P*-value, and the x-axis shows the gene ratio.

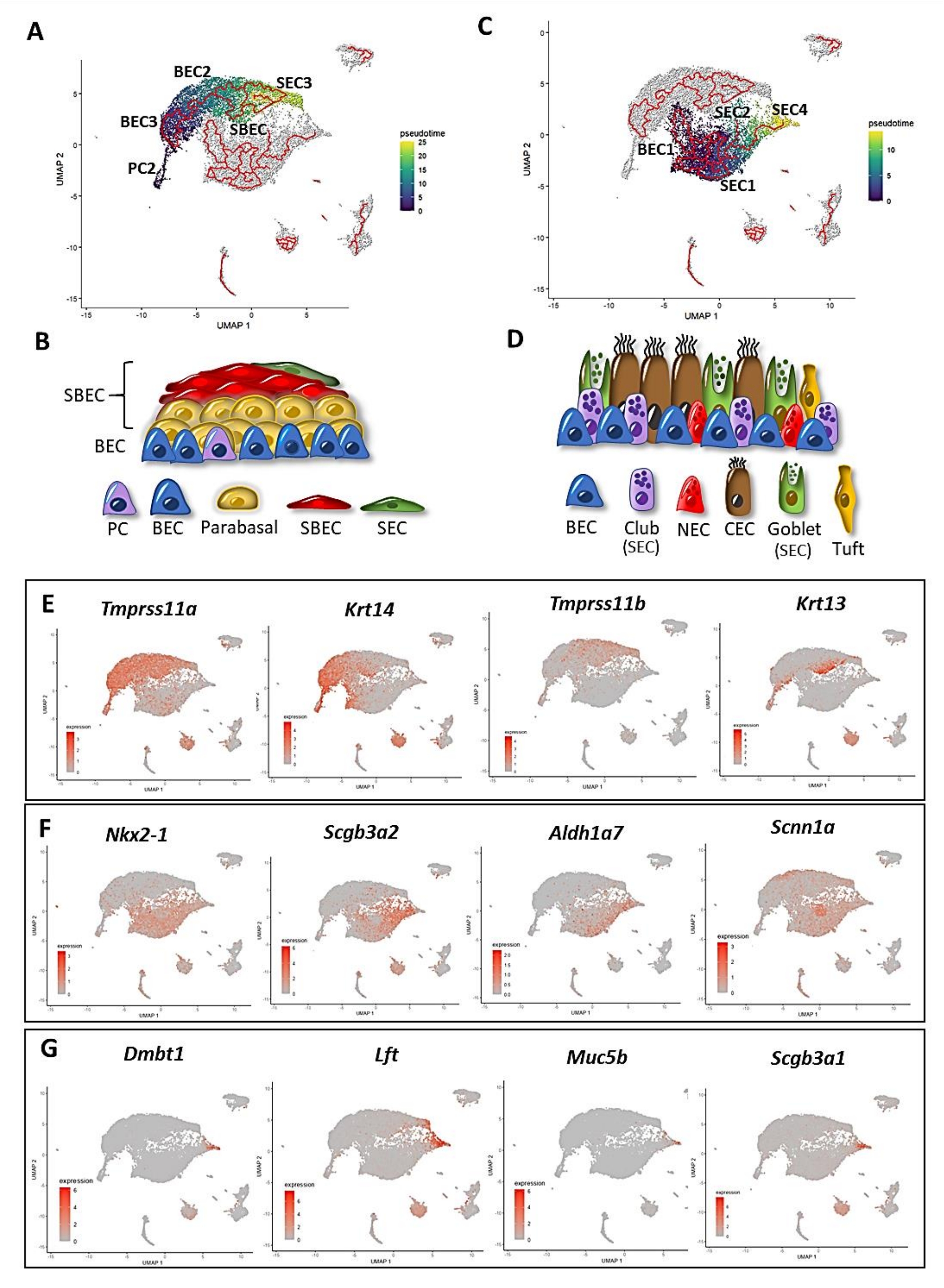

Supplementary Figure 8: Developmental trajectories within *Lrig1*⁺ epithelial cell populations. (A) Developmental trajectory of the stratified squamous epithelial lineage. Trajectory illustrates the transition from the most undifferentiated cell type, represented by BEC3 (dark blue), through the intermediate stages of BEC2 (dark green), towards the SBEC (light green), and ultimately to the most differentiated SEC3 (yellow). **(B)** Schematic illustration showing all potential cell types in SSE. **(C)** Developmental

trajectories of the pseudostratified epithelial lineage. Trajectories illustrate the transition from the most undifferentiated cell type, represented by BEC1 (dark blue), through the intermediate stages of SEC1 (dark green), towards the SEC2 (light green), or from BEC1 (dark blue) through SEC1 (dark green) towards the most differentiated SEC4 (yellow). **(D)** Schematic illustration showing all potential cell types in the pseudostratified epithelial lineage. **(E)** Genes enriched in squamous epithelial cells. **(F)** Genes enriched in pseudostratified epithelial cells. **(G)** Genes enriched in submucosal glands. Abbreviations: BEC, basal epithelial cell; CEC, ciliated epithelial cell; NEC, neuroendocrine cell; PC, proliferating cell; SEC, secretory epithelial cell; SBEC, suprabasal epithelial cell.

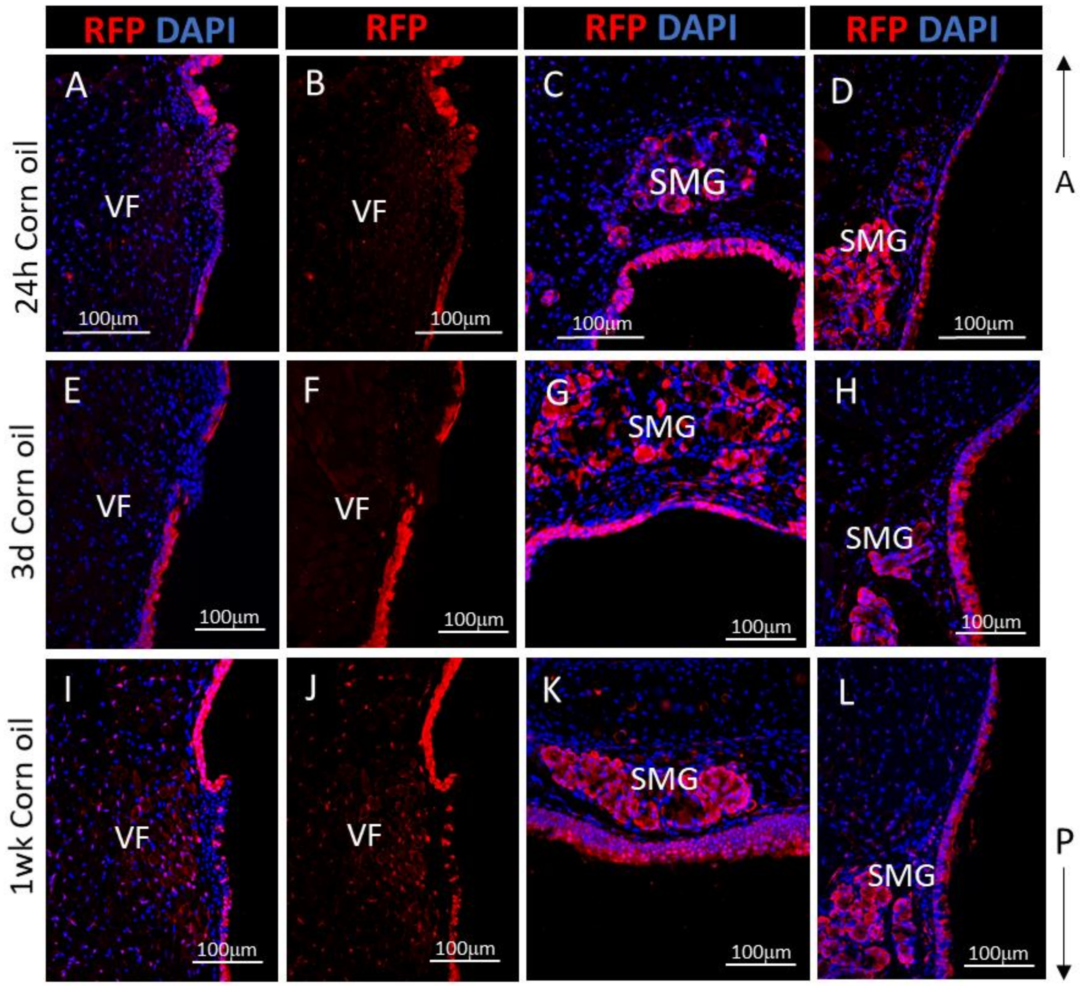

Supplementary Figure 9: Lineage tracing in *Lrig1*$^{CreERT2/+}$;*ROSA*$^{LSL\text{-}tdTom/+}$ control mice following corn oil injections. (A-D) IF staining for anti-RFP (red) and DAPI in VFs (A, B), the supraglottis (C), and the subglottis (D) in control mice 24h post corn oil injection. **(E-H)** IF staining for anti-RFP (red) and DAPI in the VFs (E, F), the supraglottis (G) and the subglottis (H) in control mice 3d post corn oil injections. **(I-L)** IF staining for anti-RFP (red) and DAPI in VFs (I, J), the supraglottis (K), and the subglottis (L) in control mice 1wk post corn oil injections. All histological analyses were performed on three biological replicates (n=3). **(**Abbreviations: A, anterior; h, hour; d, day; P, posterior; RFP, red fluorescent protein; SE, surface epithelium; SMG, submucosal glands; VF, vocal fold; wk, week.

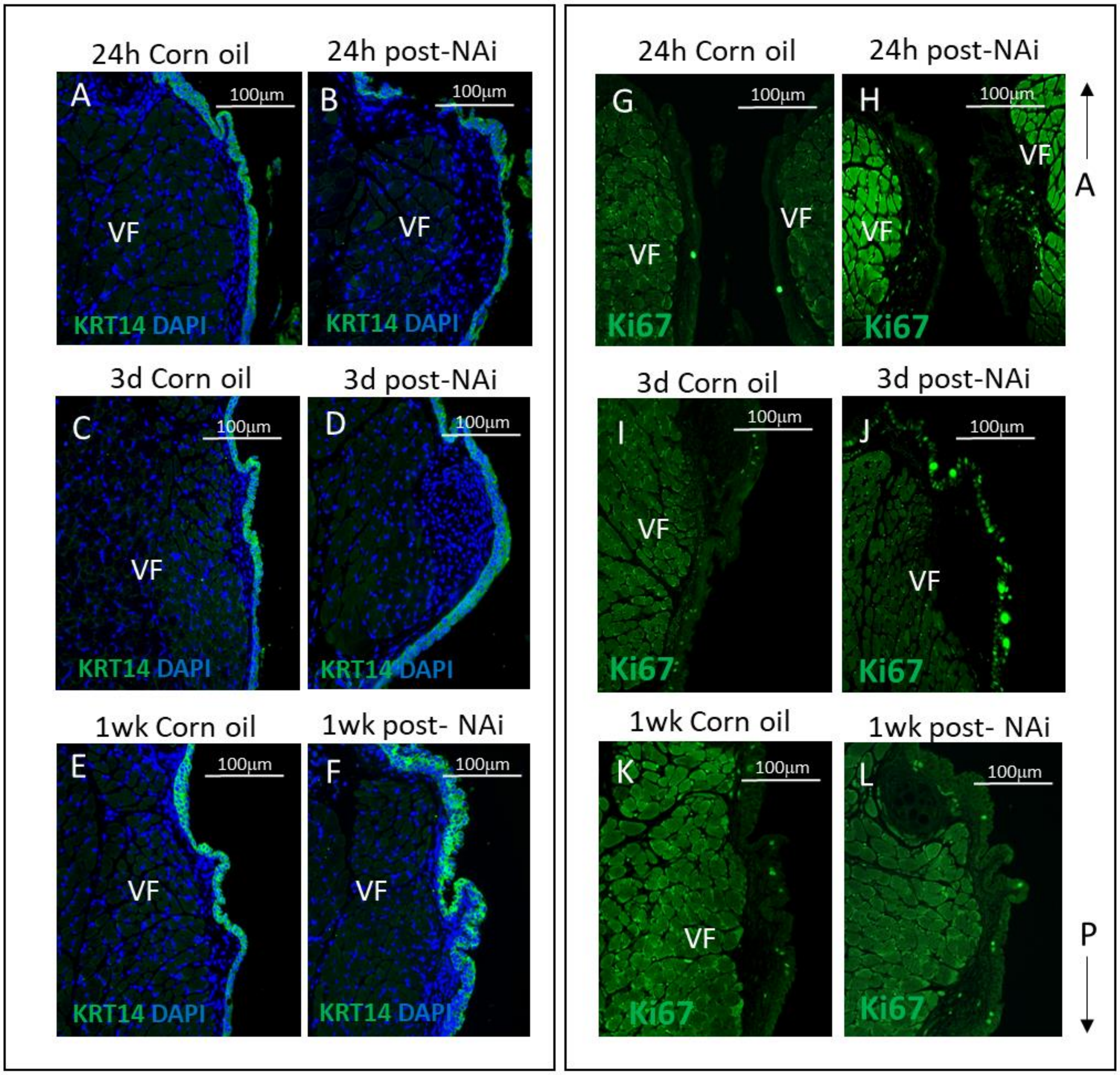

Supplementary Figure 10: Assesment of epithelial layer expansion and cell proliferation in VF epithelium in response to NA injury. (A -F) IF staining for anti-KRT14 (green) and DAPI in VFs VF mid-membranous region across timepoints in control and NA injured mice. **(G-L)** IF staining for anti-Ki67 (green) in VFs VF mid-membranous region across timepoints in control and NA injured mice. All histological analyses were performed on three biological replicates (n=3). Abbreviations: A, anterior; d, day; h, hour; mo, month; NAi, naphthalene injury; P, posterior; RFP, red fluorescent protein; VF, vocal fold; wk, week.

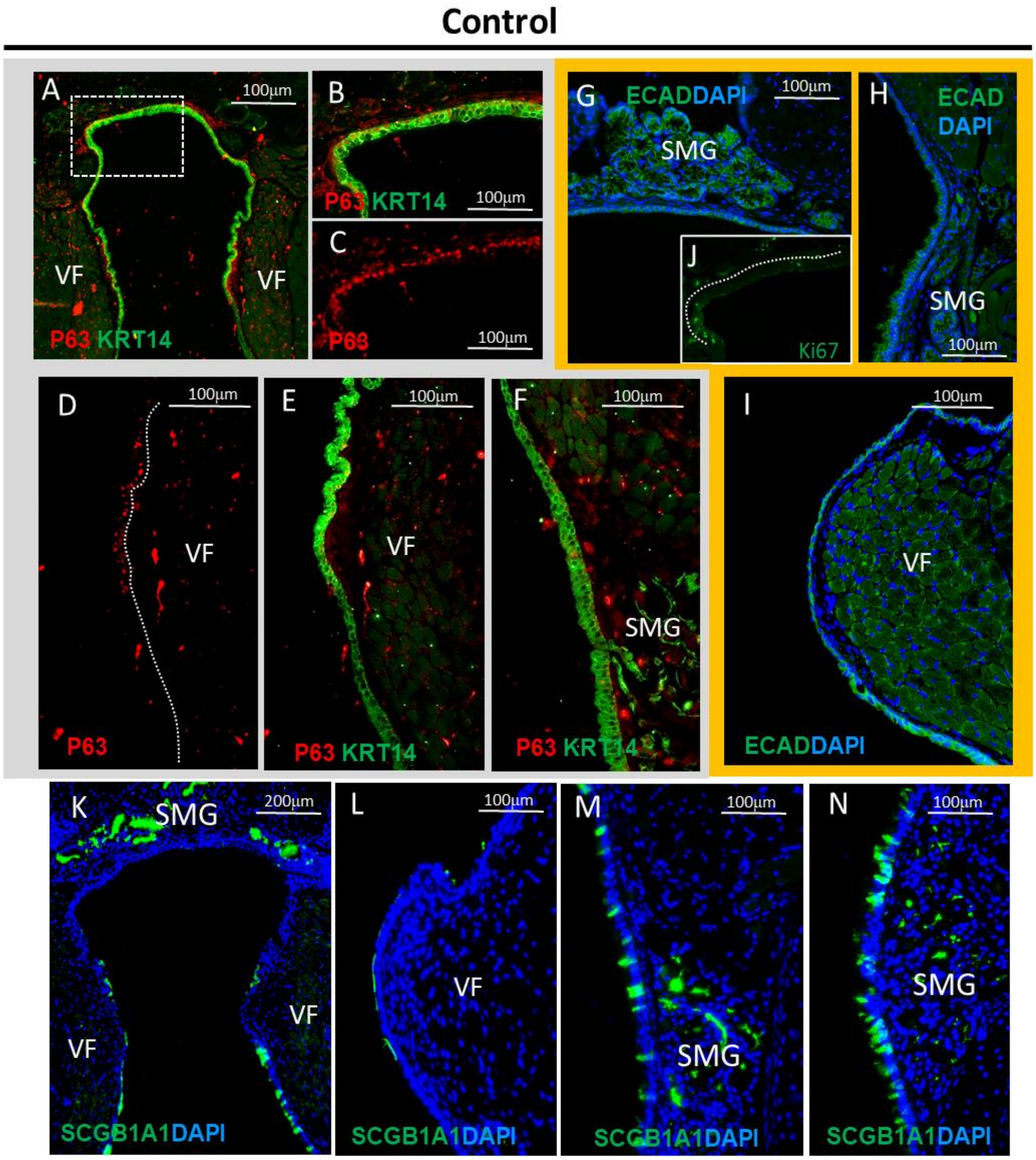

Supplementary Figure 11: IF staining for assessment of epithelial integrity and cell type distribution in control *Lrig1*$^{CreERT2/+}$ mice. (A–F) Double IF staining with anti-P63 (red) and anti-KRT14 (green) showing the morphology of the P63+ basal cell layer in the larynx (A), the supraglottis (B, C), VFs (D, E), and subglottis (F). Bracketed region in panel A is shown at higher magnification in panels B and C. A white dashed line in the panel D denotes epithelial boundary. **(G–I)** IF staining with anti-E-cadherin (ECAD; green) and DAPI (blue) illustrating epithelial compactness in the supraglottis (G), subglottis (H), and VFs (I). **(J)** Anti-Ki67 IF staining (green) in thesupraglottis, with the dashed line marking the epithelial boundary. **(K–N)** Double IF staining with anti-SCGB1A1 (green) and DAPI (blue) showing the distribution of secretory cells within the SE and SMGs in the larynx (K), VF (L), and subglottis (M, N). All histological analyses were performed in three biological replicates (n = 3). Abbreviations: SE, surface epithelium; SMG, submucosal glands; VF, vocal fold.

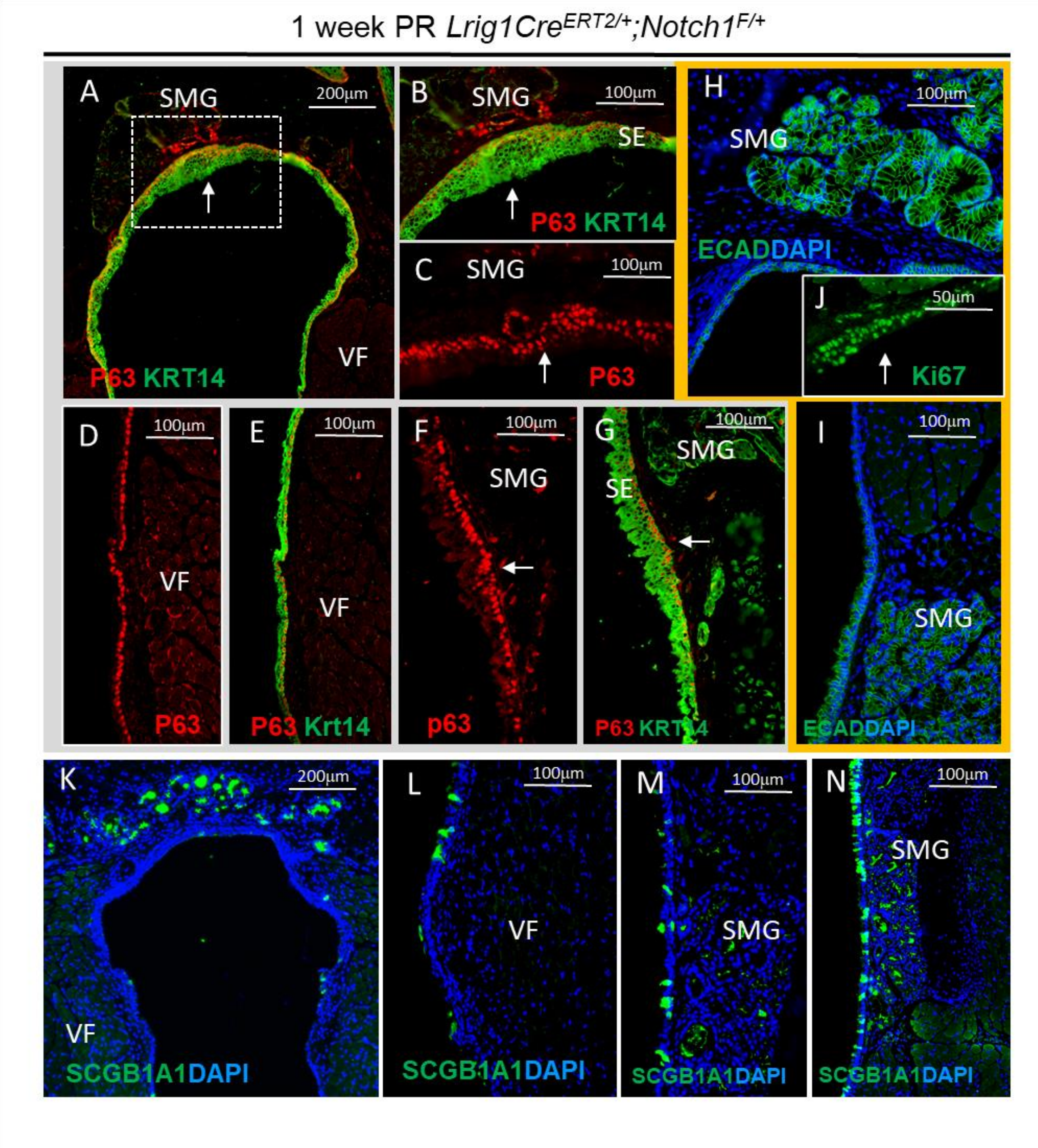

Supplementary Figure 12: IF staining for assessment of epithelial integrity and cell type distribution in heterozygous *Lrig1*$^{CreERT2/+}$ *; Notch1*$^{F/+}$ mice 1 week PR. (A–F) Double IF staining with anti-P63 (red) and anti-KRT14 (green) showing the morphology of the P63+ basal cell layer in the larynx (A), the supraglotis (B, C), VFs (D, E), and subglottic region (F). The bracketed region in panel A is shown at higher magnification in panels B and C. White solid arrows in the panels of Ab B, C F G and J denote expansion of P63+ cells. **(H, I)** IF staining with anti-E-cadherin (ECAD; green) and DAPI (blue) illustrating disruption of epithelial adherens junctions in the supraglottis (H), and compact epithelium in the subglottis (I). **(J)** Anti-Ki67 IF staining (green) in the supraglottis. **(K–N)** IF staining with anti-SCGB1A1 (green) and DAPI (blue) showing distribution of secretory cells within the SE and SMGs in the larynx (K), VFs (L), and subglottic region (M, N). All histological analyses were performed in three biological replicates (n = 3). Abbreviations: SE, surface epithelium; SMG, submucosal glands; VF, vocal fold.

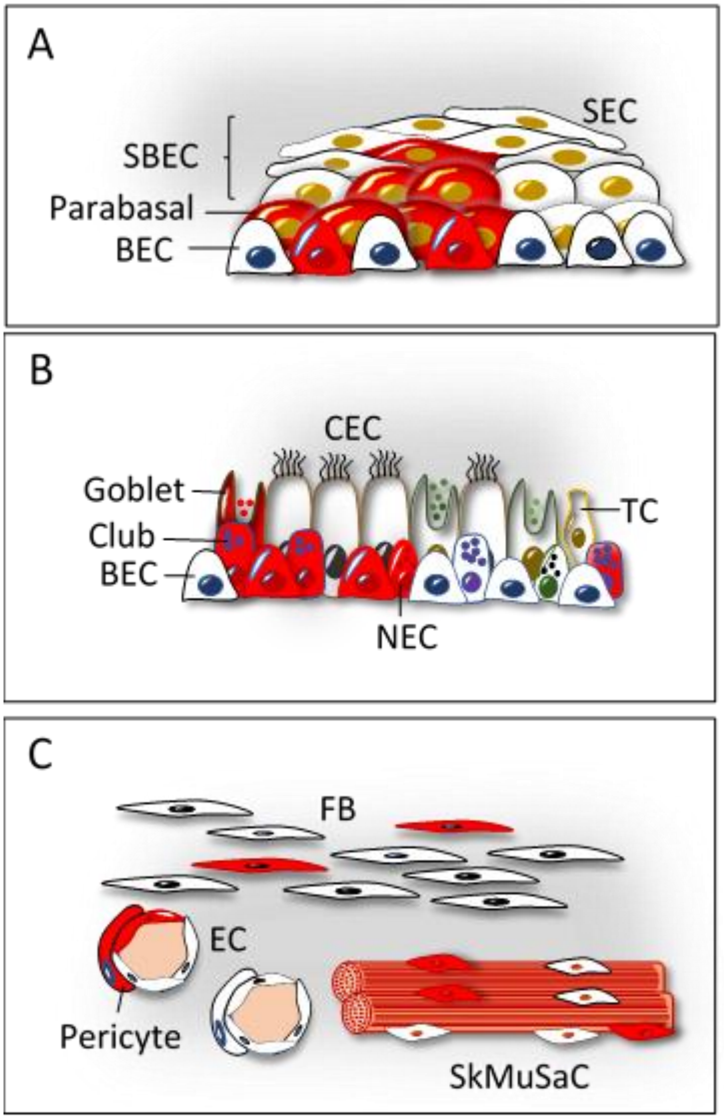

Supplementary Figure 13: Schematic illustration of *Lrig1*$^{+}$ (in red) and *Lrig1*$^{-}$ cells (in white) in SSE (A), pseudostratified mucociliary epithelium (B) and non-epithelial cell populations (C). Abbreviations: BEC, basal epithelial cell; CEC, ciliated epithelial cell; EC; endothelial cell; FB, fibroblast; NEC, neuroendocrine cell; SBEC, suprabasal epithelial cell; SEC, secretory epithelial cell; SkMuSaC, skeletal muscle satelite cell; TC, tuft cell.

Primary Ab	Species	Dilution	Cat#	Vendor
Ki67	Rb	1:150	Ab16667	Abcam
SCGB1A1	Rb	1:100	Ab213203	Abcam
P63	Ms	1:100	CM163A	Biocare Medical
KRT14	Rb	1:150	10143-1-AP	Proteintech
LRIG1	Sheep	1:50	AF7498	R&D Systems
RFP	Ms	1:100	Ab125244	Abcam
RFP	Rb	1:100	Ab15481	Abcam
MUC5B	Rb	1:100	Ab87376	Abcam
Alpha-SMA	Ms	1:100	C6198	Millilipore sigma
E-Cadherin	Rb	1:100	3195S	Cell Signaling
**Secondary Ab**				
Alexa Fluor 488	Goat	1:500	A20000	ThermoFisher
Cy3	Goat	1:200	AB_2338680	JacksonImmuno Research
Northern Lights	Donkey	1:500	NL010	R&D Systems

Gene	Primer Forward Sequence 5' → 3'	Reverse Sequence 5' → 3'
Lrig1	GCG GTC TGG CAG TAA AAA CTA TC	GTG AAA CAG CAT TGC TGT CAC TT
Notch1	CAA CAT CCA GGA CAA CAT GG	GGA CTT GCC CAG GTC ATC TA
ROSA26TdTom - WT	AAG GGA GCT GCA GTG GAG TA	CCG AAA ATC TGT GGG AAG TC
ROSA26TdTom - Mutant	GGC ATT AAA GCA GCG TAT CC	CTG TTC CTG TAC GGC ATG G

Supplementary Table 1: List of primary and secondary antibodies and primers used.
